# Supplementary material for: A portable air quality monitoring unit and a modular, flexible tool for on-field evaluation and calibration of low-cost gas sensors
Source: HardwareX. 2021 May 8;9:e00198. doi: 10.1016/j.ohx.2021.e00198 (PMC9041264; doi:10.1016/j.ohx.2021.e00198)
Supplement: Supplementary data 1 [file mmc1.docx]

**Some example of analog sensors ready to be used in SentinAir**

In the list shown below, there are some examples of sensors featured by an analog output signal that can be readily used in the SentinAir system. The list does not cover every sensor or device available on the market usable with SentinAir, because its purpose is just to provide an idea about the flexibility of the system proposed. Therefore, this is a partial list, considering that any analog sensor can be potentially used through one of the following boards: ADC Pi, LCSS adapter, or Multisensor board. Moreover, in this list, the devices having I2C, serial UART, or USB outputs are not included. All the sensors and their support boards indicated here can be used through one of the three earlier mentioned boards. For this reason, the use of each sensor of this list does not require the development of a specific driver. Further information or details about the sensors or their support board can be found on the website of their manufacturer shown in this document.

| **Sensor name** | **Measures/range** | **Support board name** | **Usable with** | **Manufacturer** |
| --- | --- | --- | --- | --- |
| *COB4* | *CO/0-1000ppm* | *Individual Sensor Board* | *Lcss adapter, ADC Pi* | *Alphasense[1]* |
| *H2SB4* | *H_2_S/0-100ppm* | *Individual Sensor Board* | *Lcss adapter, ADC Pi* | *Alphasense* |
| *NOB4* | *NO/0-20ppm* | *Individual Sensor Board* | *Lcss adapter, ADC Pi* | *Alphasense* |
| *NO2B43F* | *NO_2_/0-20ppm* | *Individual Sensor Board* | *Lcss adapter, ADC Pi* | *Alphasense* |
| *OXB431* | *O_3_/0-20ppm* | *Individual Sensor Board* | *Lcss adapter, ADC Pi* | *Alphasense* |
| *SO2B4* | *SO_2_/0-100ppm* | *Individual Sensor Board* | *Lcss adapter, ADC Pi* | *Alphasense* |
| *COA4* | *CO/0-500ppm* | *AFE sensor board* | *Lcss adapter, ADC Pi* | *Alphasense* |
| *H2SA4* | *H_2_S/0-50ppm* | *AFE sensor board* | *Lcss adapter, ADC Pi* | *Alphasense* |
| *NOA4* | *NO/0-20ppm* | *AFE sensor board* | *Lcss adapter, ADC Pi* | *Alphasense* |
| *NO2A43F* | *NO_2_/0-20ppm* | *AFE sensor board* | *Lcss adapter, ADC Pi* | *Alphasense* |
| *OXA431* | *O_3_/0-20ppm* | *AFE sensor board* | *Lcss adapter, ADC Pi* | *Alphasense* |
| *PID-A12* | *VOC/0-200ppm* | *AFE sensor board* | *Lcss adapter, ADC Pi* | *Alphasense* |
| *PID-AH2* | *VOC/0-40ppm* | *AFE sensor board* | *Lcss adapter, ADC Pi* | *Alphasense* |
| *SO2A4* | *SO_2_/0-100ppm* | *AFE sensor board* | *Lcss adapter, ADC Pi* | *Alphasense* |
| *TGS2611-E00* | *CH_4_/0-10000ppm* | *EM26* | *Lcss adapter, ADC Pi* | *Figaro [2]* |
| *FECS40-1000* | *CO/0-1000ppm* | *EM-FECS(A)* | *Lcss adapter, ADC Pi* | *Figaro* |
| *FECS41-250* | *NO/0-300ppm* | *EM-FECS(A)* | *Lcss adapter, ADC Pi* | *Figaro* |
| *FECS42-20* | *NO_2_/0-30ppm* | *EM-FECS(A)* | *Lcss adapter, ADC Pi* | *Figaro* |
| *FECS43-20* | *SO_2_/0-20ppm* | *EM-FECS(A)* | *Lcss adapter, ADC Pi* | *Figaro* |
| *FECS44-100* | *NH_3_/0-100ppm* | *EM-FECS(A)* | *Lcss adapter, ADC Pi* | *Figaro* |
| *FECS44-200* | *NH_3_/0-200ppm* | *EM-FECS(A)* | *Lcss adapter, ADC Pi* | *Figaro* |
| *FECS44-1000* | *NH_3_/0-1000ppm* | *EM-FECS(A)* | *Lcss adapter, ADC Pi* | *Figaro* |
| *FECS44-5000* | *NH_3_/0-5000ppm* | *EM-FECS(A)* | *Lcss adapter, ADC Pi* | *Figaro* |
| *FECS45-10* | *Cl_2_/0-10ppm* | *EM-FECS(A)* | *Lcss adapter, ADC Pi* | *Figaro* |
| *FECS50-100* | *H_2_S/0-100ppm* | *EM-FECS(A)* | *Lcss adapter, ADC Pi* | *Figaro* |
| *TGS5942* | *CO/0-800ppm* | *CMM5042* | *Lcss adapter, ADC Pi* | *Figaro* |
| *SP3-61* | *O_3_/0-250ppb* | *A1320301-SP361* | *Lcss adapter, ADC Pi* | *Nissha-Fis [3]* |
| *SP-61* | *O_3_/0-250ppb* | *A1320301-SP61* | *Lcss adapter, ADC Pi* | *Nissha-Fis* |
| *SB-AQ6A* | *CO_2_/400-3000ppm* | *A051020-AQ6A-01* | *Lcss adapter, ADC Pi* | *Nissha-Fis* |
| *MH-Z16* | *CO_2_/0-2000ppm* | *MH-Z16* | *Lcss adapter, ADC Pi* | *Winsen [4]* |
| *MH-Z14* | *CO_2_/0-5000ppm* | *MH-Z14* | *Lcss adapter, ADC Pi* | *Winsen* |
| *ME2-CO* | *CO/0-500ppm* | *ZE07-CO* | *Lcss adapter, ADC Pi* | *Winsen* |
| *GS+4CO* | *CO/0-2000ppm* | *AFE by Tecnosens [5]* | *Multisensor* | *DDScientific [6]* |
| *4S rev.2* | *SO_2_/0-20 ppm* | *AFE by Tecnosens* | *Multisensor* | *DDScientific* |
| *GS+4ETO* | *Ethylene Oxide/0-20 ppm* | *AFE by Tecnosens* | *Multisensor* | *DDScientific* |
| *GS+4NH3-100* | *NH_3_/0-100ppm* | *AFE by Tecnosens* | *Multisensor* | *DDScientific* |
| *GS+4NH3-300* | *NH_3_/0-300ppm* | *AFE by Tecnosens* | *Multisensor* | *DDScientific* |
| *GS+4NH3-1000* | *NH_3_/0-1000ppm* | *AFE by Tecnosens* | *Multisensor* | *DDScientific* |
| *GS+4NO* | *NO/0-250ppm* | *AFE by Tecnosens* | *Multisensor* | *DDScientific* |
| *GS+4NO2* | *NO_2_/0-30ppm* | *AFE by Tecnosens* | *Multisensor* | *DDScientific* |
| *GS+4CL2* | *Cl_2_/0-10ppm* | *AFE by Tecnosens* | *Multisensor* | *DDScientific* |
| *GS+4SO2* | *SO_2_/0-20ppm* | *AFE by Tecnosens* | *Multisensor* | *DDScientific* |
| *TDS5008* | *CO_2_/0-5000ppm* | *AFE by Tecnosens* | *Multisensor* | *DDScientific* |
| *TDS0035* | *CH_4_/0-50000ppm* | *AFE by Tecnosens* | *Multisensor* | *DDScientific* |

[1] Alphasense website: <https://www.alphasense.com/>

[2] Figaro sensor website: <https://www.figarosensor.com/>

[3] Nissha-Fis website: <http://www.fisinc.co.jp/en/>

[4] Winsen website: <https://www.winsen-sensor.com/>

[5] Tecnosens website: <https://www.tecnosens.it/en>

[6] DDscientific website: <http://www.ddscientific.com/>
