# Supplementary material for: A portable air quality monitoring unit and a modular, flexible tool for on-field evaluation and calibration of low-cost gas sensors
Source: HardwareX. 2021 May 8;9:e00198. doi: 10.1016/j.ohx.2021.e00198 (PMC9041264; doi:10.1016/j.ohx.2021.e00198)
Supplement: Supplementary data 6 [file mmc6.docx]

**A portable air quality monitoring unit and a modular, flexible tool for on-field evaluation and calibration of low-cost gas sensors**

**Author: Domenico Suriano**

**Affiliations:** ENEA - Italian National Agency for new technologies, Energy and Environment. Sustainable Development Department, Research Center of Brindisi, SS. 7, Appia, km 706, 72100 Brindisi, ITALY.

**Contact email:** *domenico.suriano@enea.it*

**Abstract:** In recent years, several research projects have investigated the use of low-cost devices for air pollution monitoring. As a result of these activities, various air quality monitoring systems have been developed following diverse approaches and methods. However, in most cases, these monitoring systems lack flexibility, extendibility, and openness. The device presented in this article provides a cost-effective, expandable, flexible, open-source solution. SentinAir has been designed to act as a portable air quality monitoring unit and also for facilitating the on-field evaluation and calibration of air quality sensors. The high grade of flexibility featuring this tool enables the data acquisition from heterogeneous kinds of devices deployed far from the laboratory facilities, even in areas featured by a weak or unstable Internet connection. Moreover, the system proposed in this article provides a reasonably easy way to integrate a wide range of sensors or devices produced by diverse manufacturers, offering a tool that is not bound to the use of a limited and fixed set of sensors or devices. The modularity approach followed in SentinAir design constitutes another key factor to consider: it allows each user to easily adapt this device to his requirements. This system is therefore capable of providing a robust framework for environmental monitoring activities to researchers or low-skilled end-users.

**Keywords**: air quality monitoring, wireless sensors, IoT, Internet of Things, sensor evaluation, sensor calibration, data logger, portable monitoring unit.

**Specifications table**

| Hardware name | *SentinAir* |
| --- | --- |
| Subject area | - Educational Tools and Open Source Alternatives to Existing Infrastructure - air quality monitoring - sensor evaluation |
| Hardware type | - Measuring physical properties and in-lab sensors - Field measurements and sensors |
| Open Source License | *GNU GPL v3.0* |
| Cost of Hardware | *78€ (basic version) - 355 € (maximum cost of typical version)* |
| Source File Repository | Mendeley Data, https://doi.org/10.17632/jfhdt44kbs.1 |

1. **Hardware in context**

One of the most concerning issues for public health is represented by atmospheric pollution [1],[2],[3]. Nowadays, air quality monitoring is mostly performed by fixed stations based on Reference Instruments (RIs) such as chemical analyzers. Although they give accurate measurements, they are quite expensive, cumbersome, and require frequent maintenance [[4]](https://www.sciencedirect.com/science/article/pii/S2352711020303022#b4), [[5]](https://www.sciencedirect.com/science/article/pii/S2352711020303022#b5), [[6]](https://www.sciencedirect.com/science/article/pii/S2352711020303022#b6). For this reason in most cases, public authorities cannot afford the deployment of a sufficient number of these stations to achieve good spatio-temporal resolutions of air pollutant maps. In order to improve this situation, the use of Low-Cost Small commercial gas Sensors (LCSSs) has become more and more popular [[7]](https://www.sciencedirect.com/science/article/pii/S2352711020303022#b5), [[8]](https://www.sciencedirect.com/science/article/pii/S2352711020303022#b7).  The LCSS term refers to a wide range of sensors or devices based on various technologies: electrochemical, resistive, Non-Dispersive Infrared Radiation absorption (NDIR) sensors, and also optical counters for Particulate Matter (PM) detection [6],[9],[10],[11]. Despite the positive aspects concerning the LCSSs, such as cheapness, portability, and low power consumption, several studies found limitations in their use. In particular, it has been proved that the reliability of data provided by the LCSSs strongly depends on the environmental conditions of the location where they are going to be deployed. Moreover, it has been demonstrated that it is of fundamental importance to calibrate them in their final deployment environment in co-location with RIs [4], [12]. The work presented in this article addresses these issues by offering a solution for the low-cost monitoring of air pollutants and also for the on-field calibration process of sensors performed in co-location with regulatory-grade instruments.

Several previous studies proposed the use of low-cost devices for environmental monitoring; among them, we can mention the monitoring unit used for air quality and malodor control presented in [13]. In this study, it was used a mix composed of in-laboratory built sensors and off-the-shelf LCSSs. The set of sensors tested in this work were able to measure NO_2_, CH_4,_ ambient temperature, and relative humidity. However, they were part of a system whose main limit was represented by a low portability grade and a fixed set of usable LCSSs.

A higher grade of flexibility was achieved by the device presented in [14]: this study proposes a battery-powered, hand-held air pollutant monitoring unit developed to measure NO_2_, CO, SO_2_, H_2_S, temperature, and relative humidity. Although this device was featured by a higher portability grade, its design was provided for the use of only one type of electrochemical sensors provided by a unique manufacturer. This limitation features also more complete monitoring systems such as EarthSense [15] and Aeroqual [16]. Both these devices offer user-friendly solutions for measuring the concentration levels related to all the typical air pollutants, but the sensors usable with these systems are limited to the only ones provided by their manufacturers. A more open approach is adopted by the Smart CItizen Station [17], which is an environmental monitoring unit designed for the detection of typical urban air pollutants along with other parameters, such as temperature, relative humidity, atmospheric pressure, and noise levels.

All the above mentioned systems do not offer a practical solution to the users who need to evaluate diverse LCSSs provided by various manufacturers, or who are in need to be supported by a compact tool able to facilitate the on-field sensor calibration process. As a matter of fact, each of the previously mentioned systems does not enable on-field data acquisitions from both LCSSs and the co-located RIs to provide ready-to-use datasets usable as input for sensor calibration processes.

The hardware presented in this article is part of the SentinAir system, which has been designed to provide both a tool that enables low-cost air quality monitoring and a compact apparatus for facilitating LCSS calibrations or evaluations. As also explained in the video released in [11] and in [18], Sentinair can perform acquisitions from both LCSSs and RIs (see figure 1) to provide output datasets that will be used as input for calibration or evaluation processes to perform through dedicated software such as the Python scikit-learn libraries [19]. This system is also designed to act as a portable monitoring unit usable in a wide range of situations, ranging from indoor environments to outdoor contexts placed in harsh or uncomfortable areas featured by a weak or unstable internet wireless link.


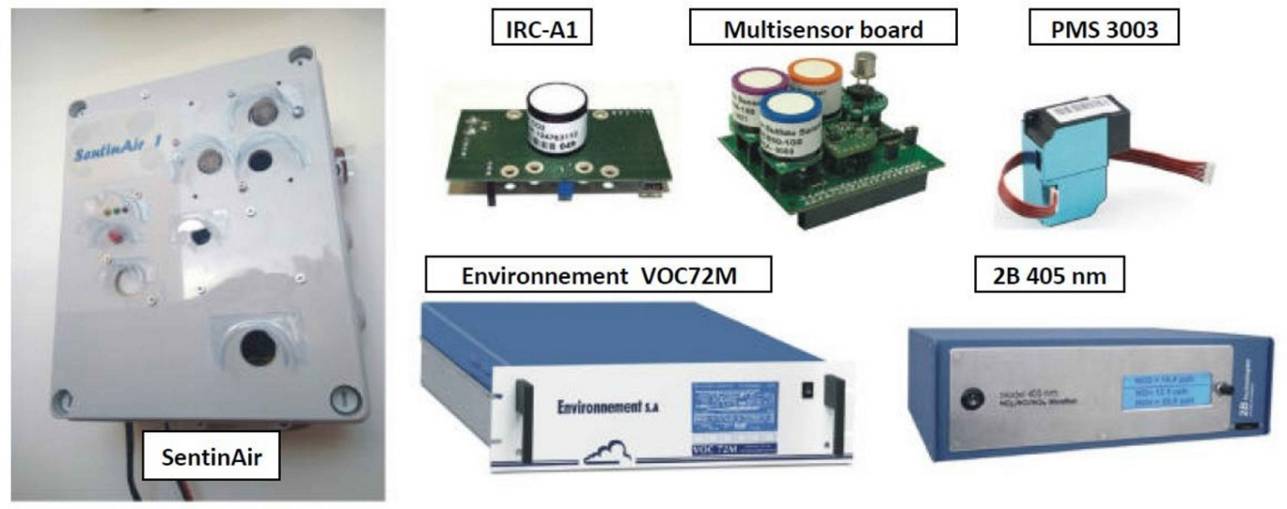


Figure 1: the SentinAir device and some examples of LCSSs and RIs which can be used with it. The typical SentinAir enclosure is a IP-56 rated plastic box, which dimensions are: 24,5 cm x 20 cm x 9 cm. Its weight depends on the sensors or devices mounted inside the box, but generally, it never exceeds 1,5 Kg.

To summarize, the SentinAir system aims to:

- Provide a compact tool that enables the use of a wide variety of sensors, devices, or instruments, allowing the users to be not bound to the use of a limited type of devices produced by a specific manufacturer or supplier,
- provide a low-cost environmental monitoring unit to use for air quality or malodours control
- provide a flexible tool for facilitating on-field LCSS evaluation and calibration
- provide an open-source solution for data acquisition from heterogeneous devices in harsh or uncomfortable environments
- provide an educational platform, thanks to its high level of openness.

1. **Hardware description.**

The concept behind the SentinAir system design can be effectively expressed as an attempt to implement a kind of “Swiss knife” usable by skilled and low-skilled users for environmental monitoring activities performed in various situations: from the research laboratories to uncomfortable environments lacking any facility. Another key aspect featuring the design of this system is represented by the potential to extend its use to activities or research areas other than air quality monitoring. The multi-purpose feature of the system proposed here is given by the particular approach followed in the design of the sensor or device management. SentinAir system considers each connected device as a “black-box” data source provided with a defined and well-known set of hardware and software interfaces. This approach enables the system to manage each sensor or instrument regardless of its nature or purpose, making feasible the use of SentinAir in various contexts and in different activities. All these factors constitute the main differences between the tool proposed in this work and other devices presented in previous studies, such as, for example, the ones illustrated in [13], [14], [15], [16], [17]. To the best of our knowledge, so far, the systems developed for environmental monitoring purposes are typically bound to the use of defined and limited types of sensors or devices, which are usually, the ones provided by the system supplier itself. In other cases, these systems are designed to be used with sensors having only a defined hardware interface, preventing the use of different devices. The SentinAir project constitutes an effort to achieve the maximum flexibility related to this aspect: each LCSS or RI having analog, USB, Ethernet, I2C, or serial digital output interface (or UART interface) can be connected to it, as also described in [10], and [11]. Moreover, the design of this system tries to provide an open-source tool that can be adapted to the requirements and needs of a wide range of users. For these reasons, the hardware modularity is the other key aspect featuring the system proposed in this work. This factor constitutes one of the main aspects of the SentinAir hardware design, which provides the option to assemble this tool in the two alternative configurations illustrated in the next subsection.

*2.1 Hardware system configurations*

The hardware proposed in this work is designed to be assembled in its basic configuration, or in its typical one. Setting up SentinAir hardware following the basic configuration scheme maximizes the ease of the assembly procedure and minimizes the implementation costs, although some functions, such as the use of devices having analog output interfaces, and also, the control from the internet, cannot be attainable. More in detail, the basic configuration of SentinAir hardware (see figure 2) is composed of:

- a *Raspberry 3 B+* board (the system mainboard),
- a class 10 SD card featured by a memory capacity of at least 2 GB (4 GB recommended),
- a 5V power source capable of providing a direct current of 3A maximum. At the moment, the system has been tested with a cheap switching ac/dc adapter,
- the sensor/device/instrument payload, which can include any device having USB, UART, Ethernet, or I2C output interface,
- an IP65 rated plastic enclosure, which enables the deployment of the system in outdoor environments.

In this configuration, the system can use each device featured by a USB, a UART, an Ethernet, or an I2C output interface. Concerning the RIs (see, for example, the VOC72M and the 2B 405nm chemical analyzers in figure 1), it must be considered that they are typically powered through their power system, and usually, they are connected through USB or Ethernet ports. On the contrary, miniaturized sensors or devices are powered through the switching adapter of the system, and they typically interface with SentinAir through USB, UART, or I2C ports.


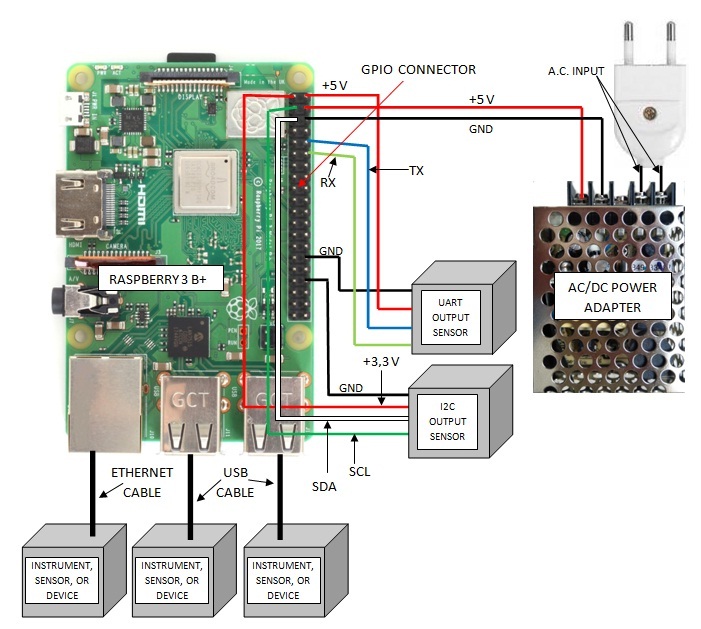


Figure 2: SentinAir basic configuration. The figure shows the wirings to connect the system components. The sensors/devices/instruments can be connected by the Ethernet or USB ports using standard cables, while sensors or devices featured by I2C, or UART outputs must follow the wiring scheme shown in the picture.

As shown in figures 26 and 27 (see section 6), the user interfaces are given by web pages and a command line shell reachable through HTTP and SSH connections by using the Wi-Fi LAN set up by SentinAir as soon as it starts up (see also [10],[11], and [20]). Unskilled or low-skilled users can interact with the system by using the web pages served by the HTTP server running on the SentinAir mainboard, while users having basic skills in the use of Linux-based operative systems can access a deeper control level of the machine through the command-line shell. This particular design of the user interfaces aims to avoid the use of dedicated applications developed for specific platforms or operative systems, although further efforts are going to be necessary to improve the user-friendliness level of the functionalities currently enabled by the command line interface system.

The typical hardware configuration (see figure 3) increases the hardware functionalities offered by the basic set-up. It includes the components featuring the basic configuration and adds other modules or subsystems. Therefore, the complete list of components concerning the hardware typical configuration is composed of:

- a *Raspberry 3 B+* board,
- a class 10 SD card featured by a memory capacity of at least 2 GB (4 GB recommended),
- a 5V power source capable of providing a direct current of 3A maximum,
- the sensor/device/instrument payload, which includes the ones usable with the basic configuration, in addition with the sensors having an analog output interface,
- an expansion board for easily connecting several sensors to be mounted inside the enclosure,
- an IP-56 rated plastic enclosure, which enables the use of the system in outdoor environments,
- an analog-to-digital (ADC) board for using devices featured by an analog output interface,
- a USB stick modem for the control of the machine from the internet,
- a push-button to easily switch-off the system,
- a LED check light system for a quick check of SentinAir status.


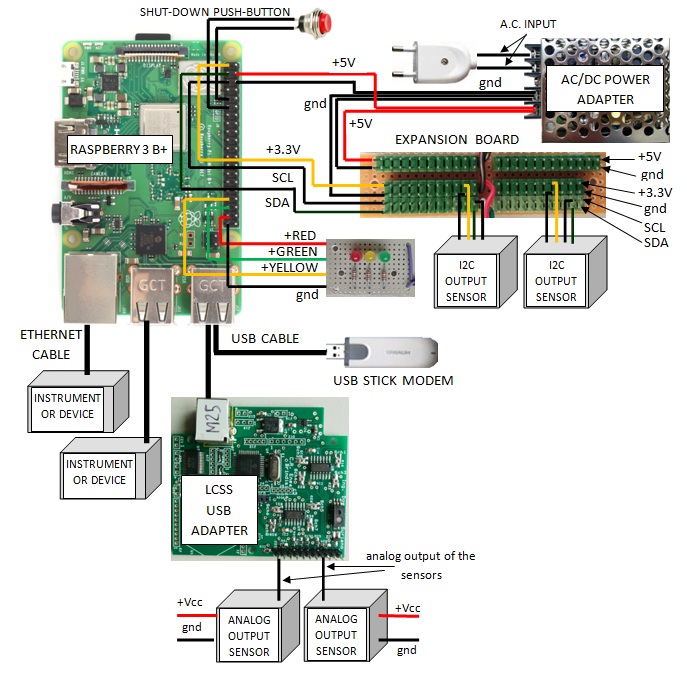


Figure 3: SentinAir hardware typical configuration. The figure shows the system components and the wirings necessary for their operation. Concerning the ADC board, the user has three options: using the ADC Pi board, the Multisensor board, or the LCSS USB adapter, which is shown in this figure. The figure also shows the expansion board designed for multiple connections of sensors or devices.

The SentinAir hardware in its typical configuration has been designed to extend the capabilities of the system. By setting up the machine following the scheme depicted in figure 3, three additional functionalities will feature the system operation. Through the USB stick modem, the users are enabled to control the system from the internet, while the check lights mounted on the lid of the enclosure enable a quick check on the current machine status. The shutdown button adds a further level of user-friendliness by allowing a quick way to properly shut down the system. However, the main functionality of the typical hardware configuration is represented by its capacity of using various sensors or devices having analog output interfaces. This feature is implemented through the integration in the system of analog-to-digital (ADC) boards. At the moment, three options are provided to implement this function: using the LCSS adapter board (as illustrated in figure 3), the ADC Pi board [21], or the Multisensor board [22].

The extreme flexibility and openness of the system imply that its power consumption and its overall weight strongly depend on the number and type of sensors mounted inside it. For this reason, it is very difficult to estimate both these parameters, although we can consider that, by using a switching power adapter providing a voltage supply of 5 Volts with a maximum output current of 3 Ampere, the maximum power consumption results to be equal to 15 Watts. However, in our experience, power consumption seldom goes beyond 6 Watts, and the total weight rarely exceeds 1,5 Kg (see also [10], and [11]).

In the following subsections, the system components featuring both the two hardware configurations will be presented in detail.

*2.2.1 The SentinAir mainboard*

The “brain” of the hardware proposed in this work is represented by a Raspberry 3B+ board (see figures 2, 3, and 4). It is a cheap mini-computer having a credit card size, featured by a Cortex-A53 (ARMv8) 64-bit microprocessor running at 1.4 GHz, and with a 1GB LPDDR2 SDRAM [23]. All the software modules and the necessary services for SentinAir operation run on the “Raspberry PI OS Lite”, which is a Linux-based operative system [24]. The Raspberry 3B+ board is featured by four USB ports, an Ethernet socket, a UART port, and an I2C bus used to interface LCSSs or RIs. The UART port can interface only one device at a time, while the bus-nature of the I2C interface enables the simultaneous connection of more devices. Its built-in Wi-Fi modem is in charge of setting up the Wi-FI LAN which acts as the main communication channel between the user and the machine. As briefly mentioned earlier, SentinAir has been designed to provide a link with the user, even if it is placed in areas where the internet wireless signal is weak or unstable. This particular feature is implemented by the on-purpose software module in charge of managing the communication channel based on e-mail sending and receiving. The system periodically attempts to connect with the remote e-mail server: if the connection succeeds, it performs a check to find the e-mails containing valid commands. If a valid e-mail is found, it will be downloaded, and subsequently, an e-mail message containing the results will be sent back. All the software modules and services installed on the mainboard are detailed in [10], [11], and [20], but they even can be summarized in:

- automatically detecting which LCSSs or RIs is connected to the system,
- reading data from the connected devices and composing the datasets stored in CSV (Comma Separated Values) files on the local SD card,
- providing a user-friendly interface through web pages served by the local HTTP server (see figure 24),
- enabling the download of datasets and system log files at any moment,
- enabling the complete control of the machine through the command line interface which is accessible by SSH connections,
- managing the communication channel based on e-mail sending and receiving,
- installing software drivers related to new devices in the pre-existing software,
- detecting the shutdown button pressing, and driving the status check lights.


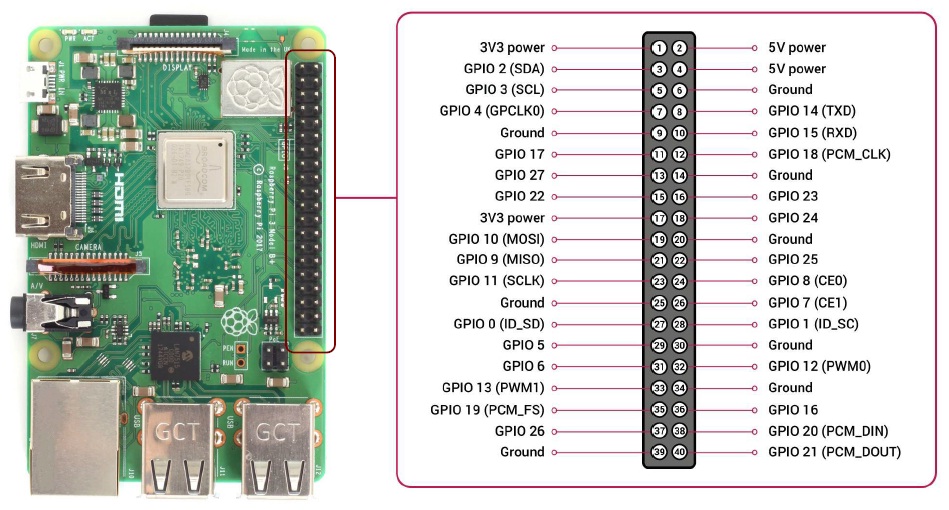


Figure 4: the system mainboard, represented by a raspberry 3 B+. Its GPIO connector is detailed on the right along with the pin function description.

*2.2.2 The system power supply*

The SentinAir power supply is in charge of providing the necessary amount of energy to operate both the hardware and the miniaturized sensors usable with it. To ensure the maximum grade of flexibility, and therefore, the use of as many sensors as possible at the same time for long periods, the system was designed to be powered through the mains electricity supply. The system components require a 5 Volts supply voltage, therefore, a power adapter must be included in the hardware design. The better option to ensure enough power availability, cost-effectiveness, and device size, is represented by a cheap AC/DC switching power adapter providing a maximum current of 3 Amps (see figure 5). Anyway, if a greater amount of power is needed, it is possible to replace the AC/DC adapter with similar models working at 5 Volts.


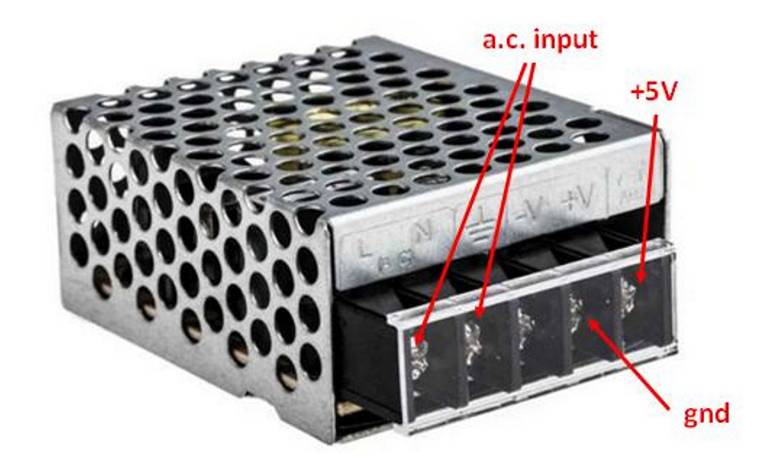


Figure 5: the ac/dc switching adapter suggested for the system power supply. Its dimensions are: 62,5 mm x 51 mm x 28 mm. The input can range from 85 to 264 Volts a.c., while its frequency can range from 47Hz to 63 Hz. The output provides 5 Volts d.c.

*2.2.3 The SentinAir enclosure*

The system flexibility enables the use of a wide range of sensors and the simultaneous handling of a considerable number of them. This factor affects the size of the enclosure necessary to arrange all the hardware, including the system payload. Nothing impedes the use of enclosures which size better fits the user's purposes, depending on the number of the sensors to mount inside it (in figure 6 there are some examples). However, from a practical point of view, to achieve the best balance combining costs, system portability, and room available for the devices mounted inside the machine, we suggest the use of a cost-effective plastic box, IP56 rated, which dimensions and shape are shown in figure 1. The suggested enclosure is a common, off-the-shelf junction box that enables to use the system in outdoor environments and also facilitates the assembly procedures of it.


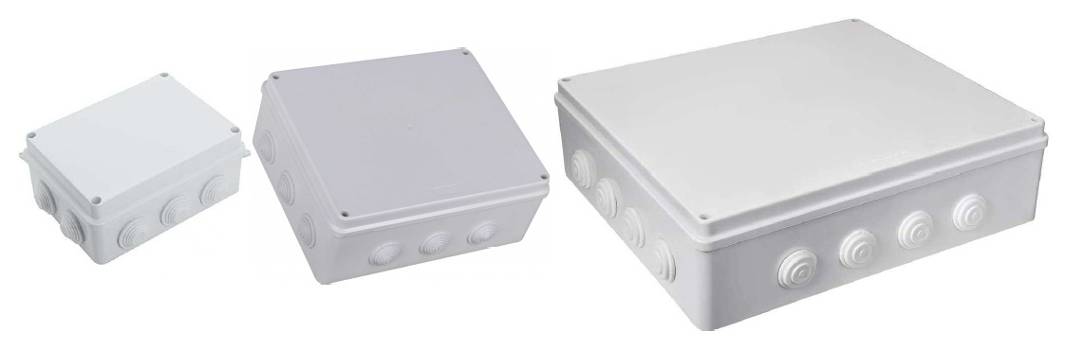


Figure6: some IP56 rated enclosures suggested for the system housing. The dimensions of the boxes shown above ranges from 150mm x 110mm x 70mm to 400mm x 350mm x 120mm.

*2.2.4 The system memory*

The operative system, the software components, and the data acquired from the LCSSs or the RIs are stored on a micro SD card. It is recommended the use of a class-4 (or superior) SD card featured by at least 4 GB.

*2.2.5 Sensors, devices, and instruments usable by SentinAir*

As introduced earlier, LCSSs or RIs usable by this system are all the ones having a USB, serial UART, Ethernet, I2C, or analog output interface. This high grade of flexibility is enabled by the system hardware and the software driver specific for each device type. The device driver set is based on software scripts written in Python and their structure is detailed in [11] and [20]. To facilitate the task of writing new device drivers, driver templates and instructions about their use are available in [20] and [25]. Anyway, a set of drivers covering a wide range of device types has been already developed (see table 1), and it is currently available in the SentinAir website repository [18]. In addition to this, to offer a higher level of friendliness, a working image of all the system software ready to use with the previously mentioned drivers can be downloaded from the repository [18] to enable an immediate use.

Table 1: device drivers currently developed for *SentinAir* and available in the repository.

| **Sensor or device** | **Device driver file** | **Connection interface** | **Supplier or manufacturer** |
| --- | --- | --- | --- |
| BME280 (temperature, relative humidity, atmospheric pressure) | bme280.py | I2C | BOSCH Sensortec |
| BH1750 (luxmeter) | bh1750.py | I2C | ROHM semiconductor |
| MCP342x on ADC Pi board (to use devices having analog output signals) | mcp342x.py | I2C | ABelectronics |
| IRC-A1 (CO_2_ sensor) | irca1.py | USB | Alphasense |
| PMS3003 (PM sensor) | pms3003.py | UART serial port | Plantower |
| Multisensor board (to use devices having analog output signals) | multisensor_board.py | USB | Tecnosens |
| 106L GO3 PRO package (CO_2_ and O_3_ monitor) | go3.py | USB | 2B technologies |
| 405nm (NOx monitor) | nox405.py | USB | 2B technologies |
| LCSS USB adapter (to use devices having analog output signals) | lcss_adapter.py | USB | Designed and built in our lab |
| CO12M (CO chemical analyzer) | co12m.py | Ethernet port | Environnement |
| AF22M (SO_2_ chemical analyzer) | af22.py | Ethernet port | Environnement |
| AC32M (NO_x_ chemical analyzer) | ac32.py | Ethernet port | Environnement |
| O342M (O_3_ chemical analyzer) | o342.py | Ethernet port | Environnement |
| VOC72M (VOC chemical analyzer) | v72m.py | Ethernet port | Environnement |

*2.2.5.1 Using sensors featured by an analog output interface*

A notable part of LCSSs available on the market is given by devices featured by analog output interfaces. In most cases, suppliers or manufacturers of such devices provide support boards for their ready use, avoiding the development of in-house electronics by the users (see figure 7). The output signals of these boards have to be converted by an ADC for enabling their elaboration and storage.


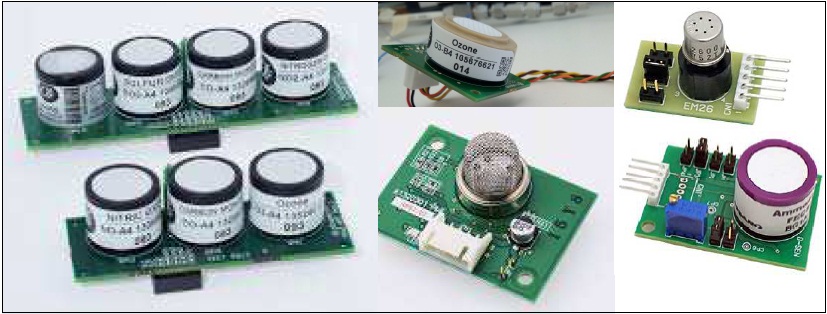


Figure 7: some examples of low-cost gas sensors featured by analog outputs and their support boards.

Unfortunately, the Raspberry board is not provided with a built-in ADC; therefore, to enable the use of them, three options are available: the LCSS adapter, the ADC Pi board, or the Multisensor board employment. Each option presents weaknesses and advantages, but all of them combined, give the necessary amount of flexibility that allows the users to select which one is the most suitable for their specific purposes. In the supplementary material, it is provided a list of sensors readily usable with one of the mentioned boards. This list is partial, and it does not include every sensor available on the market usable with them, but it just means to provide an idea about the flexibility and the openness of this system.

*2.2.5.1.1 The LCSS adapter*

The LCSS adapter (see figure 8) is an electronic board designed and developed in our laboratories aiming to provide a flexible tool that readily enables the data acquisition of LCSSs featured by analog outputs on heterogeneous systems. This board can be interfaced through its USB port, not only with the SentinAir device but also with other platforms such as Windows or Linux PCs.

The flexibility of this device also features its power supply system that offers the option to power this board through its USB socket or the 3,3 Volts Li-ion battery. These alternative power sources are managed by the hardware block built around the MCP73832 microcontroller, which is also in charge of the battery recharging. The interface between the USB and the UART port of the board main microcontroller, which is the PIC 18F4685 produced by Microchip, is implemented by the FT232RL microchip and the dedicated circuitry. The PIC 18F4685 has a built-in ADC featured with a resolution of 16 bits, which provides high accuracy to the data acquisition process. The microcontroller built-in ADC is characterized by eleven input channels: eight of them are dedicated to the acquisition of user sensor signals, while the remaining three are devoted to the reading of the battery voltage level, and the two onboard sensors measuring the temperature (through the TC1047 by MIcrochip) and the relative humidity (through the HIH5031 by Honeywell).

Concerning the communication architecture between the LCSS adapter and the interfacing systems, it has been adopted the solution given by the “master-slave” model, where the board acts as the slave device that transmits sensor data through the USB port or, through the RN42XV Bluetooth adapter which can be optionally mounted on. The Bluetooth link and the battery power supply system have been included in the board hardware design to enable the use in the stand-alone mode: in this case, the board is powered by its battery, and data are transmitted via Bluetooth to a PC or a Smartphone for their visualization and storage. In the SentinAir system, the Bluetooth option is not necessary because the LCSS adapter interfaces through the USB port. Detailed instructions about the firmware installation can be found on the SentinAir user guide [20].

One of the strong points featuring this board is represented by the high grade of flexibility that enables its use with multiple platforms and environments. On the other side, by being not available on the market, its assembly and preparation have to be carried out by the users. Unfortunately, this aspect affects its overall implementation costs, which cannot benefit from economy of scale effects.


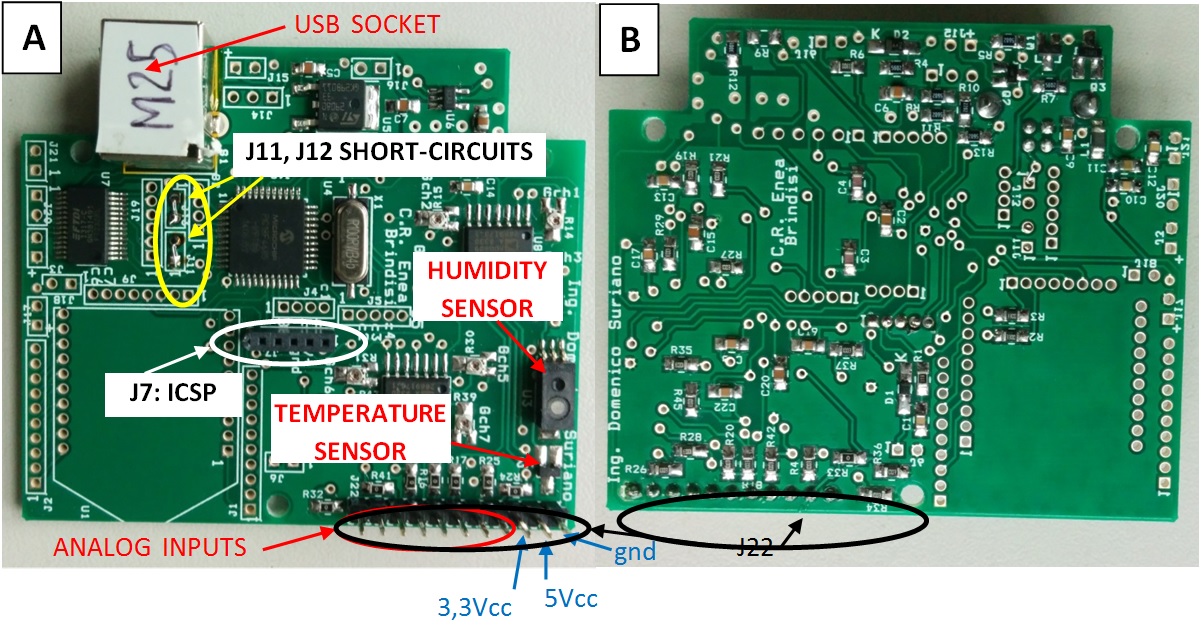


Figure 8: the LCSS adapter board. (A) top view. (B) bottom view. In the 8A picture are highlighted the connectors of relevant importance for the users. The board dimensions are 7,5cm x 6,3cm.

*2.2.5.1.2 The ADC Pi board*

The second option for interfacing analog output sensors is given by the use of the ADC Pi board supplied by ABelectronics [21]. This board is designed to work only with the Raspberry mini-computer and it is powered through the Raspberry GPIO connector (see Figure 4). The ADC Pi board is based on two MCP3424 ADCs featured by a resolution of 17 bits and each containing 4 inputs. The data converted by the ADC Pi board are transferred to the Raspberry board through the I2C bus. This board is delivered by the supplier with its two connectors unsoldered, therefore the user needs to solder them on it before its use.

The ADC Pi board is considerably less flexible than the LCSS adapter, but in contrast, by being available on the market already assembled (see figure 9), its preparation process requires very few steps. Another positive aspect concerning the use of the ADC Pi board is given by its good quality-price ratio, considering the number of available inputs and the analog-to-digital conversion resolution.


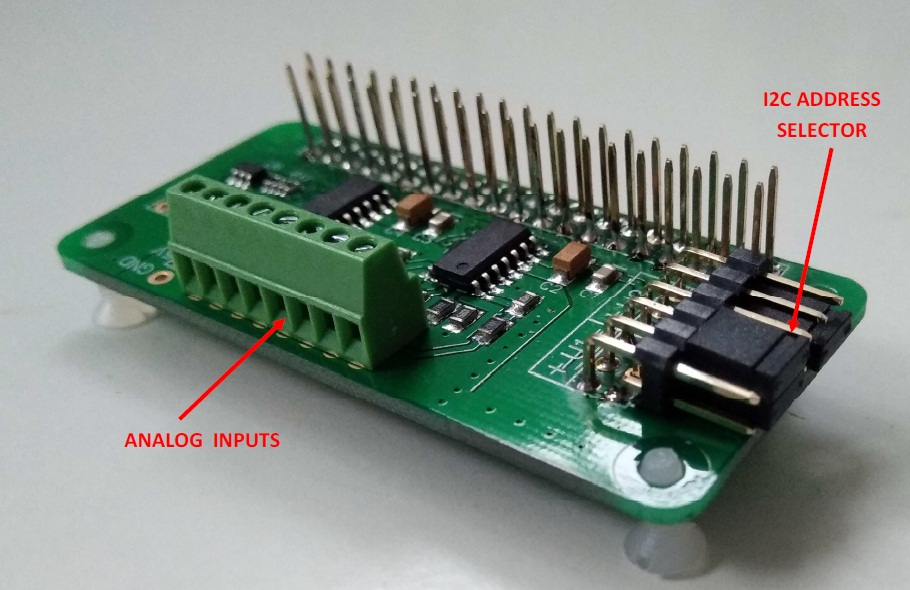


Figure 9: the ADC Pi board by ABelectronics (dimensions: 7cm x 3cm). The analog inputs from sensors can be wired through the screw terminal, while the I2C address of the board is set by the short-circuiting jumpers.

*2.2.5.1.3 The Multisensor board*

The third option for using analog LCSSs is given by the Multisensor board manufactured and supplied by Tecnosens (see figure 10). This board is designed for controlling up to six LCSSs simultaneously, moreover, it is provided with built-in sensors for measuring ambient temperature, relative humidity, and atmospheric pressure. Its USB socket acts as the interface with the master device and provides the power necessary for its operation, therefore, it must be connected to the SentinAir mainboard through the USB port.

The main weakness of this device consists in the limited number of sensor types that can be mounted on this board; moreover, LCSSs must be attached through dedicated connectors supplied only by Tecnosens. This feature represents a limit for this device because the users are bound to use only the sensor types provided with the connector developed on-purpose by the board manufacturer.

As shown on the supplier website, currently there are 14 different types of sensors available for being used with the Multisensor board. On the bright side, this device is delivered by the supplier already assembled and ready-to-use, avoiding any hardware assembly operation before its first use.


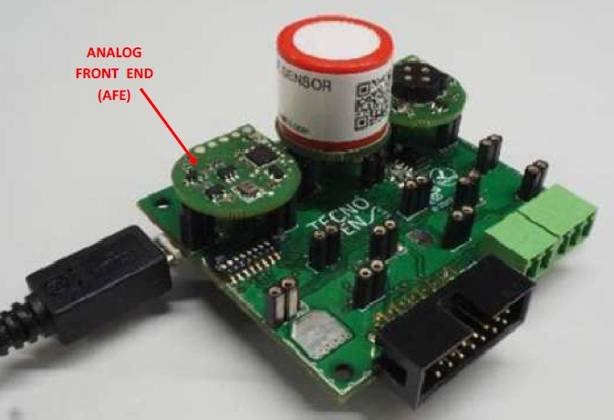


Figure 10: the Multisensor board by Tecnosens (dimensions: 6,5cm x 6,3cm). Each type of sensor must be connected through the AFE adapter (not supplied with the Multisensor board). The sensor adapters must be purchased apart from Tecnosens.

*2.2.6 The expansion board*

Miniaturized sensors or devices are typically featured by analog, UART, or I2C output interfaces. They usually need to be mounted inside the SentinAir enclosure and, in the majority of cases, such devices require a power supply voltage equal to 5 or 3,3 Volts. The typical SentinAir hardware configuration includes the expansion board that is designed to facilitate the simultaneous multiple connections of such sensors. This board is provided by header pin male connectors through which devices can be plugged for the power supply or I2C bus interfacing. Three header pin connector rows provide 5, 3,3 Volts power supply, and ground connections; in addition to them, the other two pin rows offer a quick way to connect the device SCL and the SDA signal to the mainboard I2C bus. Figures 11 and 12 illustrate the board and its terminals.


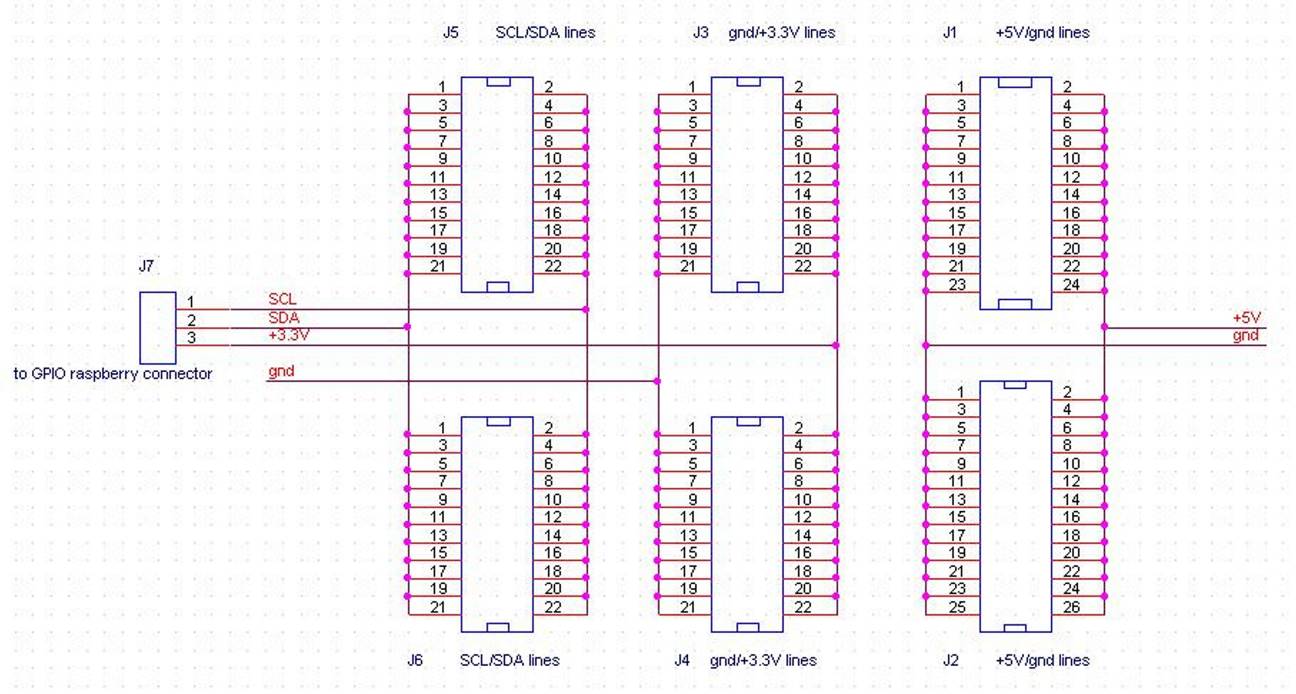


Figure 11: the expansion board schematic.


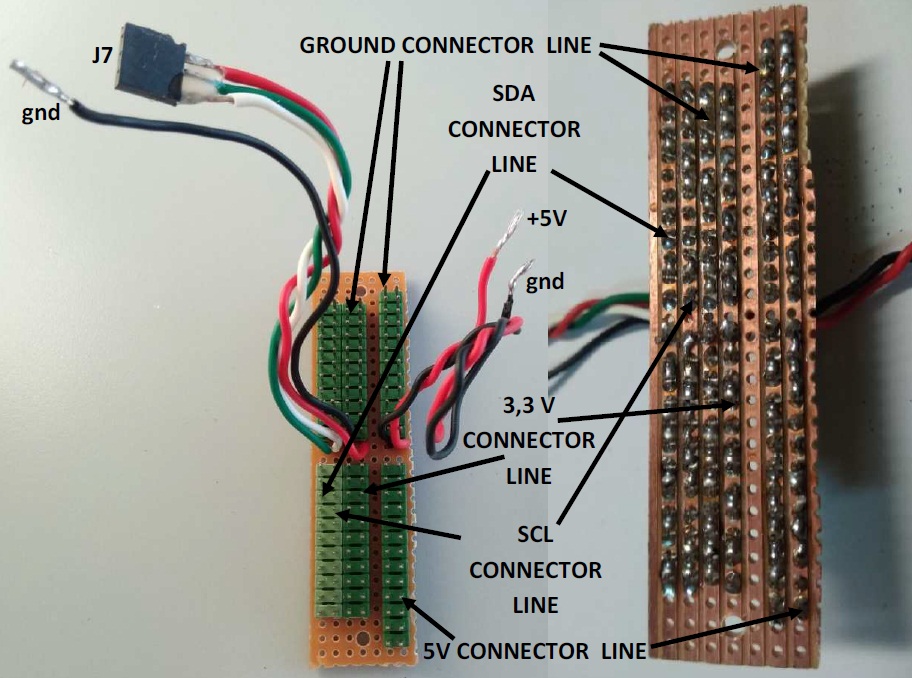


Figure 12: the top view of the expansion board on the left side, and its bottom view on the right (dimensions: 2cm x 8cm). As shown in the picture, using a bus prototype board simplifies the assembly procedures.

*2.2.7 Connecting SentinAir to the internet*

The system is connected to the internet through a USB stick modem compatible with the Raspberry operative system. The device used for the purpose is the “Huawei E303” model, but the users can replace it with similar models available on the market that are fully compatible with the Raspberry board. The control of SentinAir from the internet is feasible through the email sending and receiving system mentioned earlier, or by making reachable the SentinAir IP private address through the “IP tunneling” technology [26], [27]. For this purpose, several “IP tunneling” service companies are available on the web, each of them offering various pricing options: from free pricing plans to few Euros per month [28,29,30].

*2.2.8 SentinAir on-board user interfaces*

Three LEDs and a push-button compose the simple SentinAir on-board user interface (see figure 14). They are going to be mounted on the enclosure lid to provide a quick way to shut down properly the system and to quickly check on its status. The on-board interface has been designed to minimize its assembly steps, their difficulty, and mostly, its weight on the overall costs.

The purpose of the push-button consists in simplifying the activation of the shut-down procedure. As a matter of fact, the Raspberry operative system and all the software services must be shut down following a specific procedure. To activate this procedure the user has to access the machine operative system and launch the shut-down command, in this respect, the shut-down button offers an option for easy execution of this operation.

On the enclosure lid, it is also mounted the check light board, designed to provide ready indications to the user about the current status machine. In table 2 the LED behavior and its meaning are summarized, while figure 13 shows the board schematic.

Table 2: the onboard user interface indications given by the LED behavior.

| **LED** | **LED STATUS** | **MEANING** |
| --- | --- | --- |
| RED | OFF | System turned off |
|  | ON | System powered |
|  | BLINKING | Sensor fault (the user must see the log file to get more information) |
| GREEN | ASYNCHRONOUS BLINKING | Data exchange activity between CPU and SD memory |
| YELLOW | OFF | System active, monitoring not active |
|  | FAST BLINKING | The system is performing the port scanning to find which devices are connected |
|  | SLOW BLINKING | The shut-down procedure is going on following the shut-down button pressing |
|  | ON | Monitoring active |


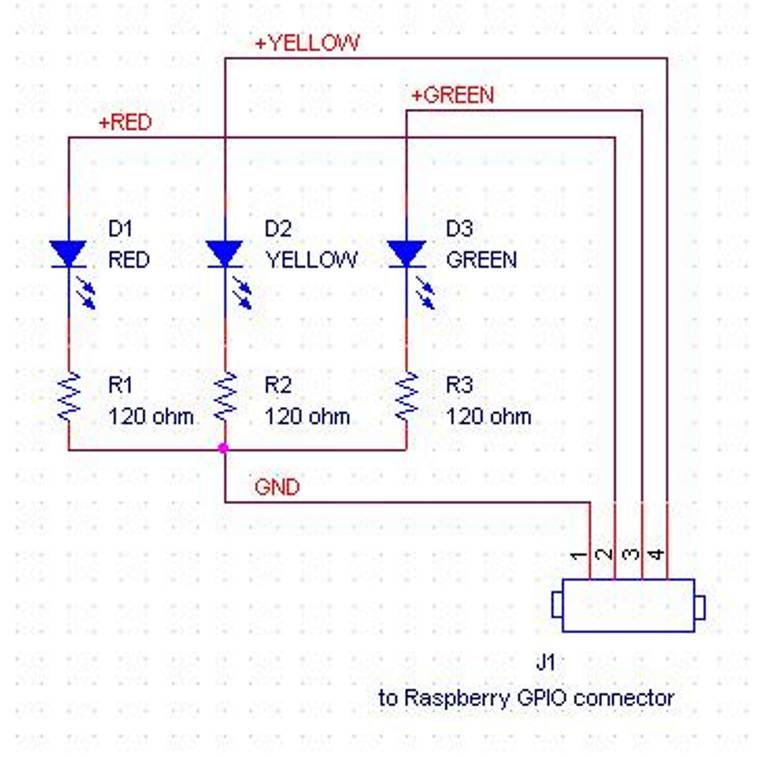


Figure 13: the check light board schematic.


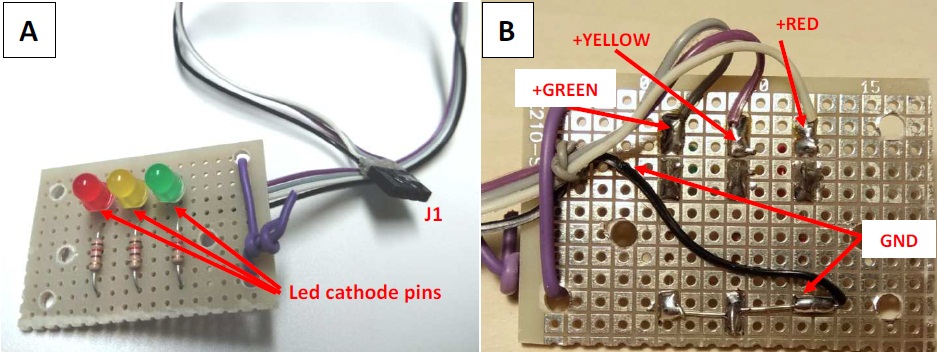


Figure 14: the system check light board (dimensions: 3cm x 4,5cm). (A) top view. (B) bottom view. The board can be built by using a prototype PCB, while the connector for the led power is a four-way female header (2,54mm pitch).

1. **Design files**

**Design Files Summary**

| Design file name/folder | File type/description | Open source license | Location of the file |
| --- | --- | --- | --- |
| */software/sentinair-S0_02022021.zip* | *Zipped System-image to install on Raspberry board* | *GNU GPL v3.0* | https://doi.org/10.17632/jfhdt44kbs.1 |
| */lcss/schematics* | Orcad 10.5 schematic files of Lcss board | *GNU GPL v3.0* | https://doi.org/10.17632/jfhdt44kbs.1 |
| */lcss/pcb project* | Orcad 10.5 pcb layout files of Lcss board | *GNU GPL v3.0* | https://doi.org/10.17632/jfhdt44kbs.1 |
| */lcss/pdf files* | Pdf files of Lcss schematic and pcb layout | *GNU GPL v3.0* | https://doi.org/10.17632/jfhdt44kbs.1 |
| */lcss/pdf files* | Firmware of Lcss board | *GNU GPL v3.0* | https://doi.org/10.17632/jfhdt44kbs.1 |
| /cad files/expansion.dxf | LibreCad file of the expansion board | *GNU GPL v3.0* | https://doi.org/10.17632/jfhdt44kbs.1 |
| /cad files/chcklights.dxf | LibreCad file of the check light board | *GNU GPL v3.0* | https://doi.org/10.17632/jfhdt44kbs.1 |
| /cad files/basic.dxf | LibreCad file of the basic hardware configuration | *GNU GPL v3.0* | https://doi.org/10.17632/jfhdt44kbs.1 |
| /cad files/typical.dxf | LibreCad file of the typical hardware configuration | *GNU GPL v3.0* | https://doi.org/10.17632/jfhdt44kbs.1 |
| /cad files/enclosure-lid.dxf | LibreCad file of the enclosure lid | *GNU GPL v3.0* | https://doi.org/10.17632/jfhdt44kbs.1 |
| /check light board | Orcad 10.5 schematic files of check light board | *GNU GPL v3.0* | https://doi.org/10.17632/jfhdt44kbs.1 |
| /expansion board | Orcad 10.5 schematic files of the expansion board | *GNU GPL v3.0* | https://doi.org/10.17632/jfhdt44kbs.1 |

1. **Bill of Materials**

**Basic configuration**

| Designator | Component | Number | Cost per unit -currency | Total cost -  currency | Source of materials | Material type |
| --- | --- | --- | --- | --- | --- | --- |
| EC400C7 | Enclosure | 1 | 10€ | 10€ | www.amazon.it | ABS |
| 137-3331 | Raspberry 3 B+ | 1 | 37.65€ | 37.65€ | it.rs-online.com | Electronic component |
| 413-673 | AC/DC power adapter | 1 | 11.27€ | 11.27€ | it.rs-online.com | Electronic component |
| TS8GUSDCU1 | SD card 8 Gb class 10 | 1 | 5.99€ | 5.99€ | www.amazon.it | Electronic component |
| 769-8736 | Plastic sheet | 1 | 13.80€ | 13.80€ | it.rs-online.com | PC |
| Total cost |  |  |  | 78.71€ |  |  |

**Typical configuration**

| Designator | Component | Number | Cost per unit -currency | Total cost -  currency | Source of materials | Material type |
| --- | --- | --- | --- | --- | --- | --- |
| EC400C7 | Enclosure | 1 | 10€ | 10€ | www.amazon.it | ABS |
| 137-3331 | Raspberry 3 B+ | 1 | 37.65€ | 37.65€ | it.rs-online.com | Electronic component |
| 413-673 | AC/DC power adapter | 1 | 11.27€ | 11.27€ | it.rs-online.com | Electronic component |
| TS8GUSDCU1 | SD card 8 Gb class 10 | 1 | 5.99€ | 5.99€ | www.amazon.it | Electronic component |
| 769-8736 | Plastic sheet | 1 | 13.80€ | 13.80€ | it.rs-online.com | PC |
| 282507365939 | USB stick modem Hawei E303 | 1 | 11.63€ | 11.63€ | www.ebay.it | Electronic component |
| 1528-2171-ND | Prototype board | 1 | 4.10€ | 4.10€ | www.digikey.com | Electronic component |
| 292-6875 | Bus prototype board | 1 | 7.50€ | 7.50€ | it.rs-online.com | Electronic component |
| 208-6677 | PCB header male 2 rows 12 ways | 4 | 2.32€ | 9.28€ | it.rs-online.com | Electronic component |
| 208-1688 | PCB header female 3 ways | 1 | 0.51€ | 0.51€ | it.rs-online.com | Electronic component |
| C5SMF-GJF-CV0Y0792CT-ND | Green led | 1 | 0.16€ | 0.16€ | Digikey.com | Electronic component |
| C5SMF-RJF-CT0W0BB1-ND | Red led | 1 | 0.12€ | 0.12€ | Digikey.com | Electronic component |
| 1516-QBL8Y30C-ND | Yellow led | 1 | 0.34€ | 0.34€ | Digikey.com | Electronic component |
| CF18JT120RCT-ND | Resistor 120 ohm | 3 | 0.08€ | 0.24€ | Digikey.com | Electronic component |
| 681-6814 | Pcb header female 4 ways | 1 | 0.80€ | 0.80€ | it.rs-online.com | Electronic component |
| 2368-54-556-2-ND | Push-button | 1 | 1.73€ | 1.73€ | Digikey.com | Electronic component |
| 765-5597 | Pcb header female 2 ways | 1 | 0.73€ | 0.73€ | it.rs-online.com | Electronic component |
| Electronic board for analog signal conversion (option 1) | ADC board | 1 | 28.33€ | 28.33€ | www.abelectronics.co.uk | Electronic component |
| Electronic board for analog signal conversion (option 2) | LCSS USB adapter | 1 | 65.48€ | 65.48€ | See bill of materials below | Electronic component |
| Electronic board for analog signal conversion (option 3) | Multisensor board | 1 | 240€ | 240€ | www.tecnosens.it | Electronic component |
| Total cost (option 1) |  |  |  | 144.18€ |  |  |
| Total cost (option 2) |  |  |  | 181.33€ |  |  |
| Total cost (option 3) |  |  |  | 355.85€ |  |  |

**LCSS USB adapter**

| Designator | Component | Number | Cost per unit -currency | Total cost -  currency | Source of materials | Material type |
| --- | --- | --- | --- | --- | --- | --- |
| C1, C10,C12 | KEMET C0805C104Z5VACTU | 3 | 0.08€ | 0.24€ | digikey.com | Electronics |
| C2, C5, C6, C7, C8, C15, C20 | KEMET C0805C106K8PACTU | 7 | 0.13€ | 0.91€ | digikey.com | Electronics |
| C3, C4 | KEMET C0805C220J5GACTU | 2 | 0.08€ | 0.16€ | digikey.com | Electronics |
| C9, C13, C14, C16, C17, C18, C19, C21, C22 | KEMET C0805C103K1RACTU | 9 | 0.08€ | 0.72€ | digikey.com | Electronics |
| C11 | KEMET C0805C475K9PACTU | 1 | 0.17€ | 0.17€ | digikey.com | Electronics |
| D1 | BAV20WS-TP | 1 | 0.17€ | 0.17€ | digikey.com | Electronics |
| D2 | PMEG3010EH | 1 | 0.28€ | 0.28€ | digikey.com | Electronics |
| J7 | Harwin M22-7130542 | 1 | 1.16€ | 1.16€ | digikey.com | Electronics |
| J22 | Harwin M20-9773646 | 1 | 0.91€ | 0.91€ | digikey.com | Electronics |
| L1 | Murata LQW31HN33NJ03L | 1 | 0.62€ | 0.62€ | digikey.com | Electronics |
| Q1 | IRLML9301TRPBF | 1 | 0.31€ | 0.31€ | digikey.com | Electronics |
| Q2 | DMP2160UW-7 | 1 | 0.23€ | 0.23€ | it.rs-online.com | Electronics |
| Q3 | ZXT11N20DFTA | 1 | 0.5€ | 0.5€ | digikey.com | Electronics |
| R1 | RC0805FR-0710KL | 1 | 0.08€ | 0.08€ | digikey.com | Electronics |
| R2, R3, R6 | CRGCQ0805F330R | 3 | 0.80€ | 0.24€ | digikey.com | Electronics |
| R4, R5, R7, R8, R11, R13 | CRG0805F56K | 6 | 0.08€ | 0.48€ | digikey.com | Electronics |
| R9, R10 | CRGCQ0805F22K | 2 | 0.08€ | 0.16€ | digikey.com | Electronics |
| R12 | CRG0805F3K0 | 1 | 0.08€ | 0.08€ | digikey.com | Electronics |
| R14, R15, R22, R23, R30, R31, R38, R39 | EVM-2NSX80B55 | 8 | 0.74€ | 5.92€ | digikey.com | Electronics |
| R16, R17, R24, R25, R32, R33, R40, R41 | RC0805FR-070RL | 8 | 0.08€ | 0.64€ | digikey.com | Electronics |
| R18, R19, R20, R21, R26, R27, R28, R29, R34, R35, R36, R37, R42, R43, R44, R45 | RC0805FR-07100KL | 16 | 0.08€ | 1.28€ | digikey.com | Electronics |
| S1 | 748-0866 | 1 | 1.00€ | 1.00€ | it.rs-online.com | Electronics |
| PCB | LCSS PCB | 1 | 4.88€ | 4.88€ | pcbcart.com | PCB |
| U2 | TC1047AVNBTR | 1 | 0.51€ | 0.51€ | digikey.com | Electronics |
| U3 | HIH-5031-001S | 1 | 22.63€ | 22.63€ | digikey.com | Electronics |
| U4 | PIC18F4685T-I/PT | 1 | 7.45€ | 7.45€ | digikey.com | Electronics |
| U5 | LD29080DT33R | 1 | 0.99€ | 0.99€ | digikey.com | Electronics |
| U6 | MCP73832T-3ACI/OT | 1 | 0.46€ | 0.46€ | digikey.com | Electronics |
| U7 | FT232RL-REEL | 1 | 3.74€ | 3.74€ | digikey.com | Electronics |
| U8, U9 | AD8609ARZ | 2 | 4.20€ | 8.40€ | digikey.com | Electronics |
| X1 | AS-10.000-20-3030-SMD-TR | 1 | 0.17€ | 0.17€ | digikey.com | Electronics |
| Total cost |  |  |  | 65.48€ |  |  |

1. **Build Instructions**

5.1 Potential safety hazards

The necessary safety measures must be applied when soldering, wiring, cutting, or drilling.

5.2 Assembling the LCSS adapter board

The bare LCSS board PCB or the assembled PCB can be implemented by uploading the “Bill of materials”, the hardware design, or the “Gerber” files to any PCB assembler. However, this option represents a cost-effective solution only if consistent batches of this device are ordered. On the contrary, electronic components need to be manually soldered. This operation must be carried out by soldering the components listed in the “Bill of materials” on their silkscreen designator locations indicated on the PCB. All the passive components are ‘0805’, or bigger, sized. The last operation is the short-circuiting of J11 and J12 (see figure 8).

5.3 Assembling the ADC Pi board

The ADC Pi board is optional to the LCSS board. It can be purchased from the manufacturer [21] already assembled except for the Raspberry connector, the I2C address selector, and the analog input screw terminal. Detailed instructions about their soldering operations can be found on the manufacturer's website [21].

5.4 Assembling the expansion board

The procedure to assembly the expansion board is composed of the following steps:

1. Cut the bus prototype PCB to obtain a size of 2cm x 7.8cm, then drill two holes (3mm of diameter) at its ends as shown in figure 15,
2. Solder the PCB male headers on each of the five lines (SDA, SCL, +5V, GND, +3,3V, GND) as shown in figure 15,
3. Solder six wires 10cm long for each line (SDA, SCL, +3,3V, GND, +5V, GND) on the PCB as shown in figure 12, then solder a 3-way female PCB header (2,54mm pitch) to the other ends of the SDA, SCL, +3,3V wires (see figure 12).


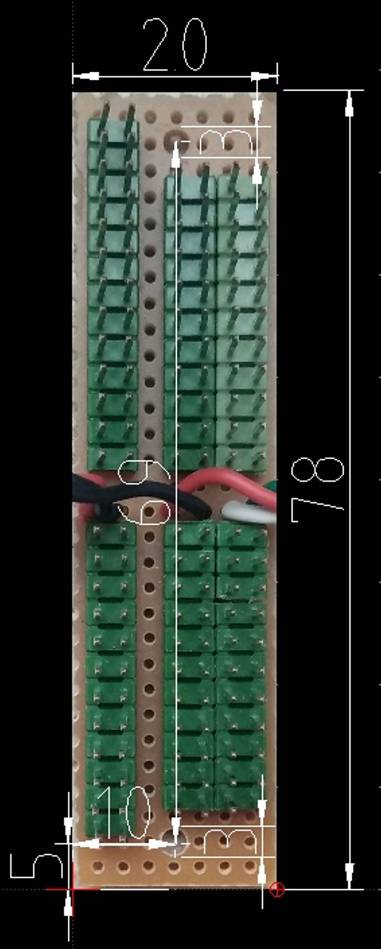


Figure 15: the expansion board, its size, hole position, and diameter. Dimensions are in millimeters.

5.5 Assembling the system check light board

The procedure to assembly the system check light board is composed of the following steps:

1. Cut a prototype PCB piece sized as in figure 16. Drill two holes having a diameter of 3mm.
2. Solder the three 120 ohm resistor, and the three LEDs in the positions shown in figure 17 and following the electrical wirings depicted in figure13. Mind to the right position of the LED cathodes (see figure 13, 14, 16),
3. Solder four wires 20 cm long to the three LED anodes and to the common terminal of resistors (see figure 14), then solder a four-way female PCB header (2,54mm pitch) to the other wire ends.


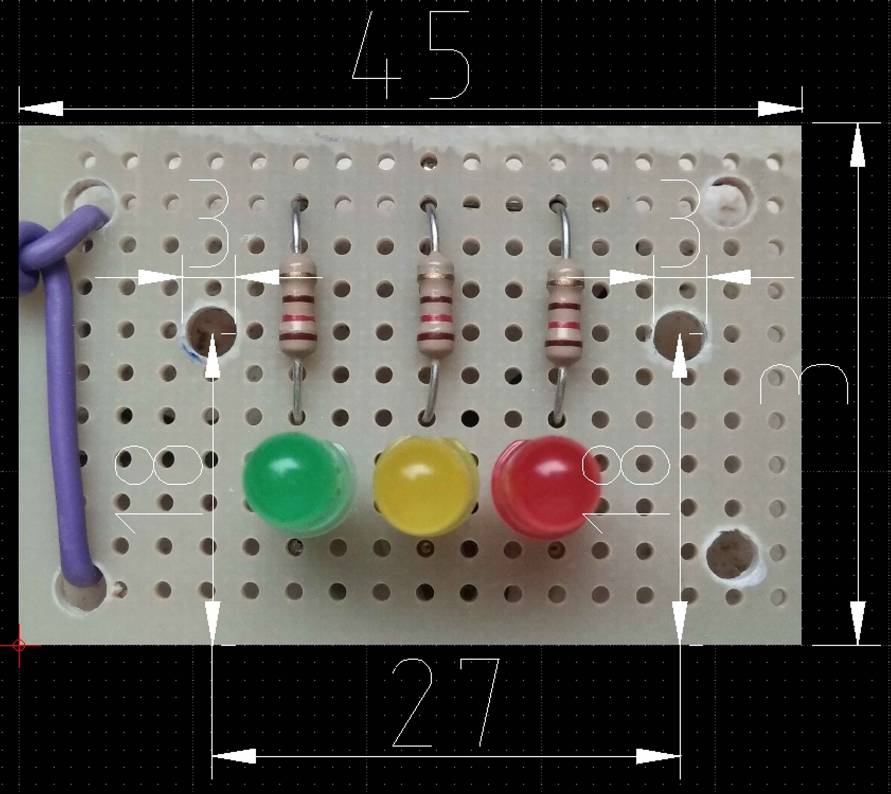


Figure 16: the check light board. Dimensions are in millimeters.

5.2 Assembling the basic or the typical hardware configuration

The user can choose the enclosure which most suits his application because the hardware components displacement inside it does not present critical points or issues. Therefore, each user can decide how and where to arrange them by just ensuring that the sensor sensing surfaces are properly exposed to the external environment. However, in this section, a tested layout is proposed just to provide a template that can be customized to best fit user specific requirements.

The enclosure proposed here is represented by a junction box (see section 2.2.3 and figure 6) IP56 rated, which size is 24cm x 19cm x 9cm. All the components can be placed on a plastic baseplate where mounting holes must be drilled for the fixing. Therefore, for assembling the hardware in its basic or typical configuration, the plastic baseplate must be drilled following the scheme depicted in figure 17, and subsequently, the hardware components (to form the basic or the typical configuration) can be fixed using screws and bolts. The check light board, the push button, and the LCSS adapter (if the user decides to employ it) must be fixed to the enclosure lid at his backside, drilling the holes in the positions suggested by the scheme of figure 18. Once the baseplate and the enclosure have been prepared, and the components have been fixed, they must be connected following the indications of figures 2, 3, 4, 5, 8,12, 14.


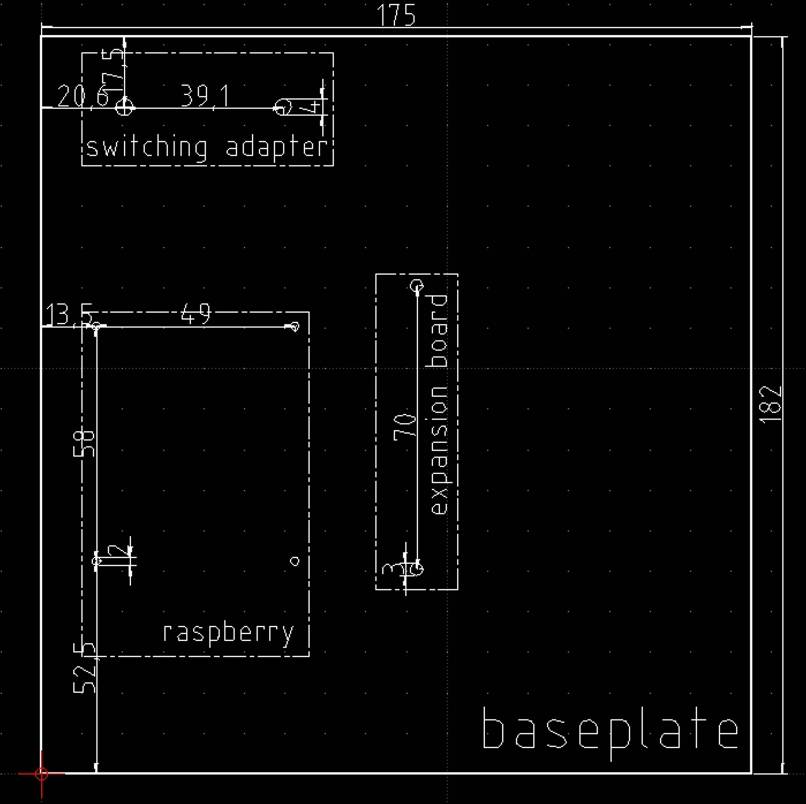


Figure 17: the dimensions of the baseplate to insert in the enclosure, the holes to drill, their position and size, and the component profiles indicated by dashed lines.

The final step to finish the hardware assembly is represented by the fixing of plastic hoods to protect the enclosure openings from the rain. The hoods are obtained by cutting with scissors some strips roughly sized 2cm x 5cm from recycled plastic bottles. By using a hot glue gun, they must be fixed above each opening of the lid as shown in Figure 20. This system is an effective and extremely low-cost way to protect from the rain the electronics inside the enclosure, as proved in figure 19. The final aspect of the assembled hardware is shown in figure 20. The approach to follow for arranging and fixing the sensors into the enclosure is similar to the procedure exposed so far. In general, it is possible to attach the sensors to the lid through screws as shown, for example, in the case of the LCSS adapter. Holes for screws and openings for exposing the sensor sensing surface to the external environment must be drilled in the right position depending on the features of each sensor or device that the user decides to mount in SentinAir. Figure 21 shows, as an example, the way for installing the IRC-A1 sensor, the BME280, the BH1750, and other analog sensors onto the enclosure lid.


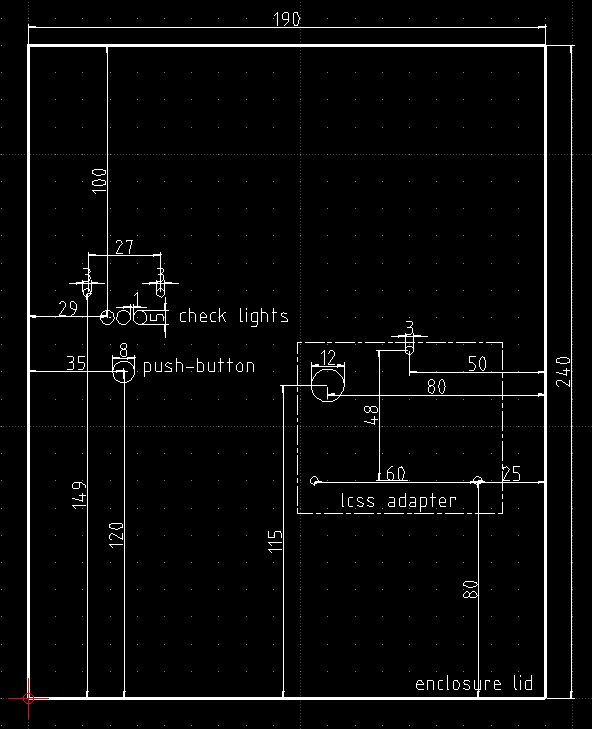


Figure 18: the enclosure lid top view schematic. In this picture are shown the holes to be drilled for fixing the check light board, the push-button, and the optional LCSS adapter (indicated by the dashed lines).


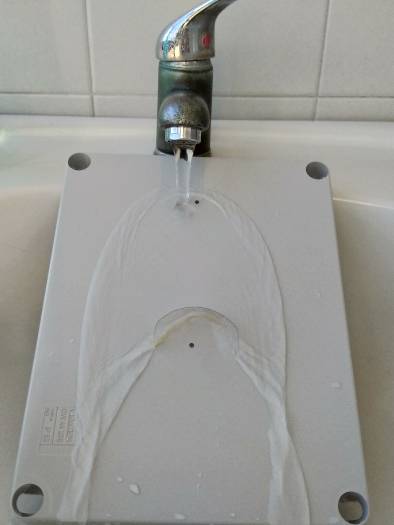


Figure 19: a simple test to prove the effectiveness of hoods obtained by cutting strips from recycled plastic bottles and fixed through hot glue on the enclosure lid.


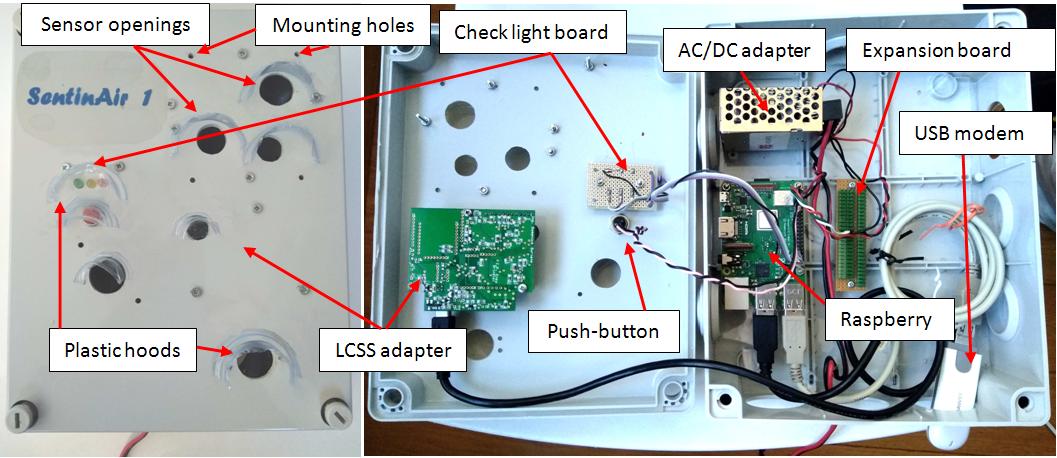


Figure 20: the final aspect of the assembled hardware. On the left, it is shown the top view of the enclosure lid, while on the right it is shown the displacement of the component inside the enclosure.


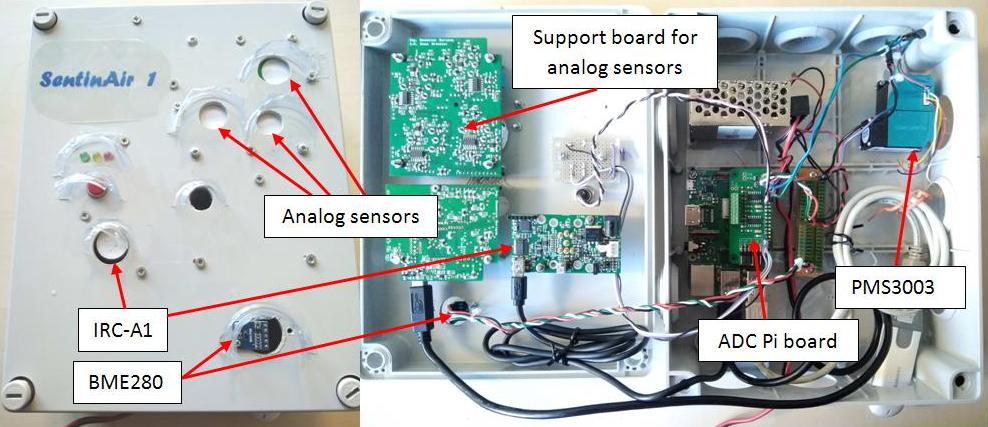


Figure 21: one of the many possible ways to arrange various sensors: the PMS3003 is fixed on the baseplate, while the IRC-A1, the BME280, and the support board for the COB4, NO2B43F, and the OXB431 by Alphasense are fixed back the lid. Holes for exposing the sensors to the external environment along with mounting holes have been drilled in the enclosure lid in the proper position, depending on the specific sensor.

5.3 Installing the required software

The software required to operate the hardware is composed of the operative system for the mainboard, and the additional modules, services, and libraries. There are two optional ways to set up all the software: the first one consists of downloading, installing, and configuring every single component one at a time, the second one is given by downloading and installing a system-image already working. The first procedure requires several steps that are detailed in the SentinAir user guide [20], while the second procedure is way more simple and is composed of the following steps:

1. Download the system-image by using the on-purpose link available in the SentinAir repository [18], and “unzip” the file to get the system-image file,
2. Write the system-image on an SD card featured by 4 GB, through the open-source tool “Etcher” [31], which runs on Windows, Mac, and Linux platforms; other similar tools can be used for the purpose.

At this point, the software for the mainboard is ready for use with all the main functionalities. The machine is configured with the default name “sentinair-S0”, user and password are “pi” and “raspberry”. The user can already use the device with this default configuration, but optionally he can customize it by changing some key data: password, machine name, date, and time. Date and time are read from the internet if the hardware is configured in its typical configuration, otherwise, the user must manually set them. Changing the credentials, machine name, and setting date and time can be done by plugging a USB keyboard and a monitor through the HDMI port, or by connecting through the WiFi LAN set up by SentinAir as soon as it gets started. In this second case, the user must establish an SSH connection to the IP address “192.168.4.1” by using client programs such as, for example, “PuTTY” [32], or similar applications. The credentials to use are “pi” (user), and “raspberry” (password). Once the log-in has been done, to change the password it must be launched the command *'sudo passwd'*, and subsequently, the new password must be inserted two times. To change the date and time, the command is '*sudo date –s “dd month(first three letters) yyyy hh:mm:ss"'* (for example: sudo date –s “12 Feb 2021 11:26:21”). Conversely, the procedure for changing the machine name is composed of three steps:

1. Launching the built-in program called “raspi-config” by typing *'sudo raspi-config'* and selecting the voices '2 Network options' and 'N1 Hostaname' [33]. Then insert the new machine name. It is mandatory that the name must have a “hyphen” character inside, for example: “sentinair-S1”.
2. Modifying the file called *“hosts”* by using the text editor “nano”. To open this program, it must be typed the command *'sudo nano /etc/hosts'*. Then, if the new name is, for example, “sentinair-S1”, the string *“127.0.0.1 sentinair-S0”* must be changed in*“127.0.0.1 sentinair-S1”*. After this operation, the file must be saved and closed by pressing “CTRL+O” and “CTRL+X” (see figure 22),
3. Rebooting the system by launching the command '*sudo reboot*'.


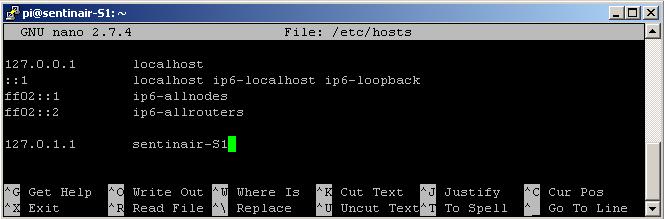


Figure 22: the screen showing the file “/etc/hosts” opened by the text editor called “nano”. To save the file, the user must press the “CTRL” key and the “o” key; while, to close the file, press the “CTRL” key and the “x” key.

If the system is assembled in its typical hardware configuration, the user can enable the module designed for the machine control through e-mails (see sections 2.2.1 and 6.4). By default, after the first installation, this module is not enabled because the file *“mail-config.sentinair”* does not contain any valid e-mail account. To enable the module, the user must modify this file by inserting valid credentials of his e-mail account. This operation can be summarized in:

1. Opening the file *“mail-config.sentinair”* through the text editor *“nano”* by typing *'sudo nano /home/pi/sentinair/mail-config.sentinair'*
2. Inserting the required information as shown in figure 23,
3. Saving and closing the file as explained earlier,
4. Rebooting the system as explained earlier.


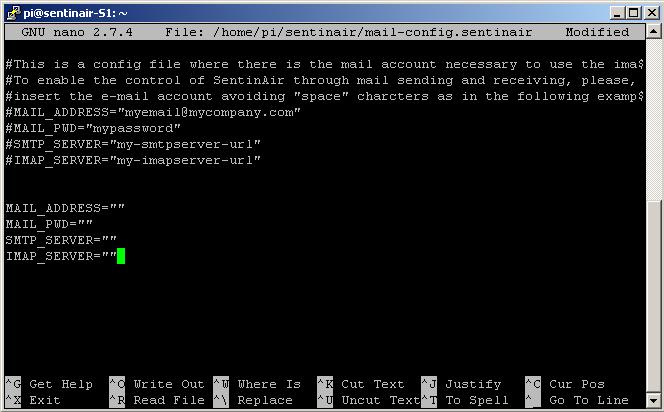


Figure 23: the “*nano*” text editor showing the file “*mail-config.sentinair*”. In the file are included instructions and examples about how to correctly insert data for enabling the e-mail communication module.

Further details concerning the system software can be found in the SentinAir user guide [20].

1. **Operation Instructions**

The first necessary operation to use the SentinAir hardware is the connection of, at least, one of the devices listed in Table 1. Subsequently, the system gets started by plugging the power cable to a suitable power source featured by an alternated current ranging from 85 to 264 Volts at a frequency ranging from 47Hz to 63 Hz (which could be the standard electricity wall socket in most countries). Before plugging the power cable, it is highly recommended to close the device enclosure to avoid electric shock risks. After few seconds, the green check light starts to asynchronously blink, indicating the operative system setting up. Subsequently, the yellow check light starts to fastly blink, showing that the system port scanning is underway for enabling the automatic acknowledgment and connection of the sensors or instruments. Thus, the red light turns on to show that the device is correctly powered. After this stage, if the system has been started for the first time, or previously it was shut down in the " active monitoring" mode, a new monitoring session starts, and the yellow light remains on. When a new monitoring session gets started, three new files containing the measurement datasets are created in the folder *“/var/www/html/data”* stored in the system SD card. The files contain respectively the measurement data, their hourly, and daily averages automatically computed by the system. They are named respectively following the formats: “*sentinair-SX_yyyy-mm-dd_hh-mm-ss.txt”,* “*sentinair-SX_yyyy-mm-dd_hh-mm-ss_hourlymeans.txt”,* “*sentinair-SX_yyyy-mm-dd_hh-mm-ss_dailymeans.txt”;* where *“yyyy-mm-dd_hh-mm-ss”* is the current date and time*.*

During the system launch, the machine sets up a WiFi LAN which enables the access to the system for its control or data downloads. Once the user gets access to the WiFi LAN, it is possible to check the device activity by establishing a connection to the fixed IP number of the machine, which is “192.168.4.1” for default, through a standard HTTP connection using a common web browser (see figure 24). The web server running on the system mainboard enables the user to:

- Get information about the last measurement, and read the sampling rate of the current monitoring session,
- View the time series of all the measurements,
- Download or view all the datasets present in the system SD card,
- Download or view the log files.


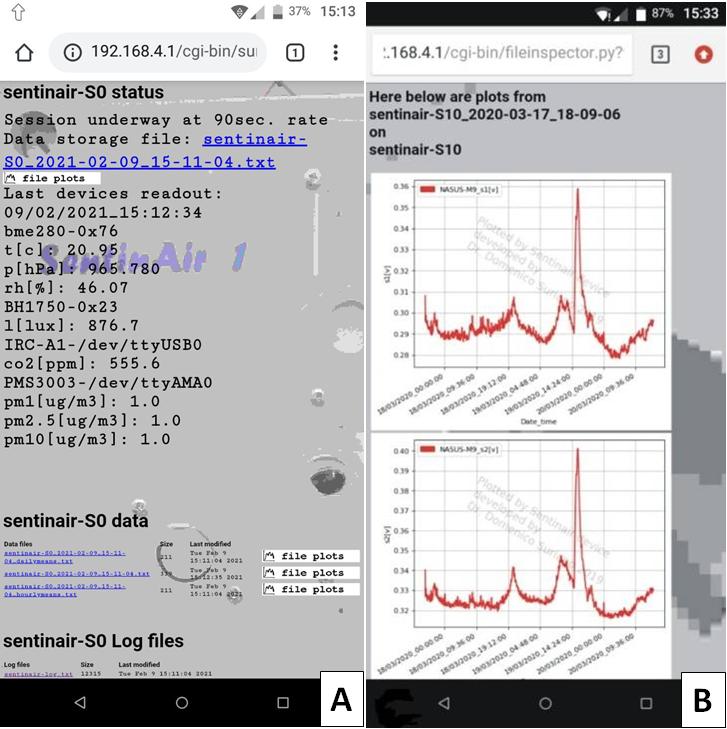


Figure 24: the data included in the web pages: (A) on the main page is shown the current sampling rate, the current storage file, the last measurements, all the data and log files present in the SD card. By clicking on the file links, it is possible to view or download them. By clicking on the “file plots” button, the user opens the page showing the time series graphs related to each of the measures stored in the related file.

*6.1 Advanced control of the system through the user command line interface*

The complete control of the system is enabled by launching the on-purpose user command-line interface (see figure 25). To activate it, the necessary procedure is constituted by the following steps:

1. Connecting to the WiFI LAN of the system,
2. Establishing an SSH connection by using clients such as, for example, the “PuTTY” application, or similar programs,
3. Accessing the system by using the user name (“pi”), and password (default password is “raspberry”),
4. Typing the command: *'cd /home/pi/sentinair'*
5. Typing the command *'python3 sentinair.py'*

Once the user has activated the interface, he is enabled to completely control the system by launching commands through the command line (see figure 26). The list of the commands available for controlling the device is summarized in table 3.

Table 3: commands available from the command line user interface

| **Command function** | **Command syntax** | **Example** | **Effect** |
| --- | --- | --- | --- |
| Starting a new monitoring session | s,seconds | s,60 | A new monitoring session gets started. Every 60 seconds, device measurements data are read and stored in a file |
| Stopping a measurement session | b | b | Stops the current measurement session |
| Retrieving information about the system status | i | i | Displays information, such as the devices currently connected to the system, the sampling rate of the measurement session, or if the system is on standby. |
| Retrieving information about the devices connected to the system | c | c | The system returns the information about the connected devices: identity, units of measurements, current measurements |
| Asking for the list of available commands | h | h | Displays a brief manual where are shown the commands |
| Scanning the ports and connecting the devices plugged into *SentinAir* ports | s | s | The system scans all the ports (USB, Ethernet, etc.) and recognizes if one of the installed devices is plugged into |
| Quit the program | q | q | Quits the program |


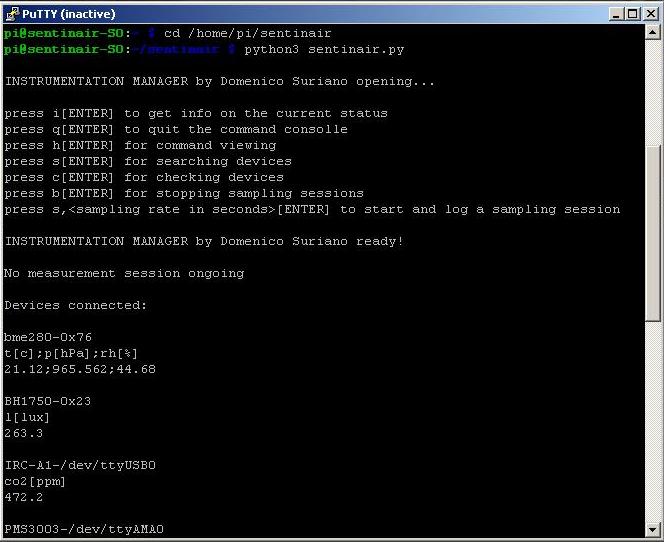


Figure 25: the command-line interface for the advanced control of the system. The commands to open the program and their responses are shown in this figure.


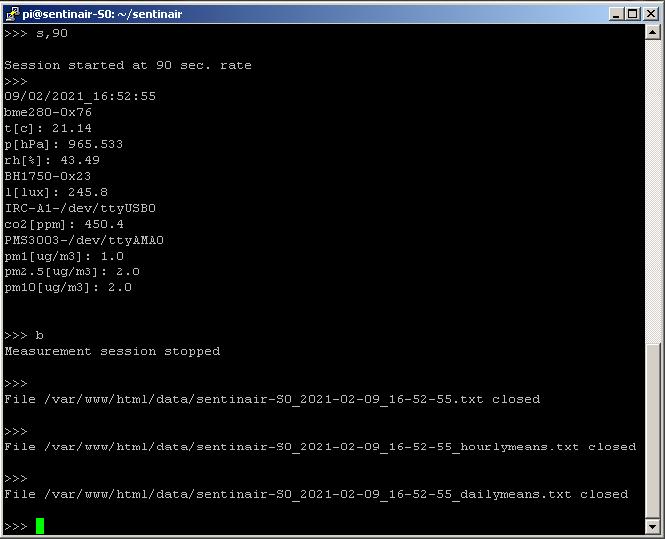


Figure 26: some examples of command-line interface commands. This figure shows the launch of a new monitoring session (“s,90” command) having a sampling rate of 90 seconds, and its break (“b” command).

*6.2 Managing the software drivers of sensors or instruments*

As explained earlier, each device to be used with SentinAir needs a specific software driver to be installed in the system. Only the devices which driver is already installed will be recognized and connected. To carry out the installation, the uninstallation, or just for checking which drivers are currently installed, the user must run the on-purpose command-line program by following the steps from 1 to 4 indicated in section 6.1, and then, by typing the command *'python3 sentinair_system_installer.py'* as shown in figure 27. The commands available and their purpose are listed in table 4, while figure 27 provides some examples of their use.

Table 4: list of commands available for the installer manager program.

| **Command function** | **Command syntax** | **Example** | **Effect** |
| --- | --- | --- | --- |
| Device driver installation | i,driver_name.py | i,pms3003.py | Installation of the driver for the device pms3003 |
| Device driver uninstalling | u,driver_name.py | u,pms3003.py | Uninstallation of the driver for the device pms3003 |
| Checking the currently installed drivers | c | c | Displays a list of the drivers currently installed |
| Modifying the current path of the system manager module | m | m | Writes in the file "manager_dir.sentinair" the new path of the manager system module |
| Asking for the commands list available | h | h | Displays a brief manual where are shown the commands |
| Quit the program | q | q | Quits the programs |


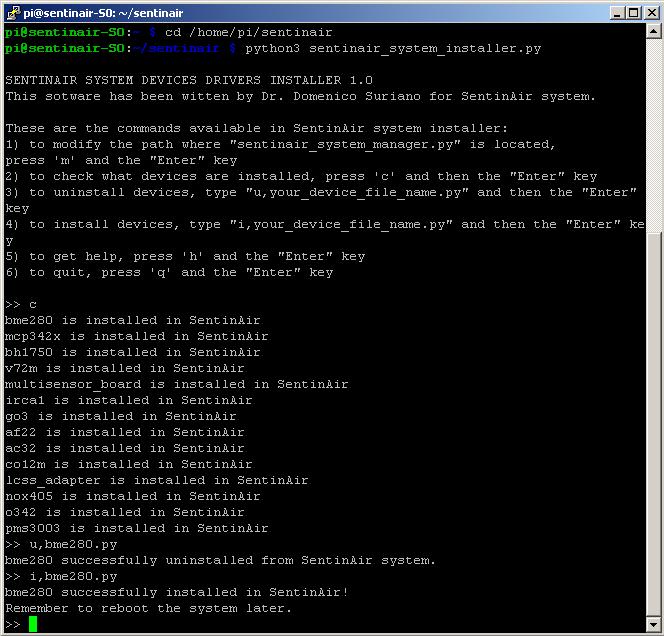


Figure 27: examples of commands available for the installer manager program. In this picture, it is shown the “c”, the “i,driver_name.py”, and the “u,driver_name.py” commands, along with the system responses.

*6.3 Connecting the system to the internet*

The system in its typical configuration can be optionally connected to the internet by plugging a USB stick modem into one of the USB ports on the mainboard (see section 2.2.7). The modem gets automatically recognized if it is compatible with the Raspbian operative system. The user has two options to control the device through the internet: the first one consists in using one of the various “IP tunneling” services available on the web [28,29,30]. In this case, he must follow the instructions provided by the selected service. The second option is represented by the e-mail command system.

*6.4 Using the e-mail command system*

To control SentinAir through the e-mails, the user must send e-mails to the address specified in the file *“mail_config.sentinair”*. The e-mail subject must contain the string "*device_ID system command*",

or the string "*device_ID SentinAir command*" (pay attention to the letter case), where “device_ID” is the suffix in the machine name (if the machine name is, for example, “sentinair-S0”, the “device_ID” will be “S0”). In the first case, the command written in the e-mail body will be executed by the operative system, while, in the second case, it will be executed by the SentinAir software. In both cases, the command must be in the format 'do: command' (for example: 'do: i', or 'do: ls –l', see figure 28 and 29). In case the e-mail contains a command for the SentinAir software, it must be one of those included in table 3. Conversely, commands for the installation manager are not provided for the e-mail communication system. Through this communication channel, it is possible to download files from the device by sending a particular command that will be executed by the operative system. Its format is 'fget: /folder_path/filename'; for example, to download a data file, the e-mail body must contain the string '*fget: /var/www/html/data/sentinair-S10_ 2021-02-09_15-11-04.txt'*. The e-mails containing the responses will be sent in few minutes, depending on the wireless internet signal stability and strength.


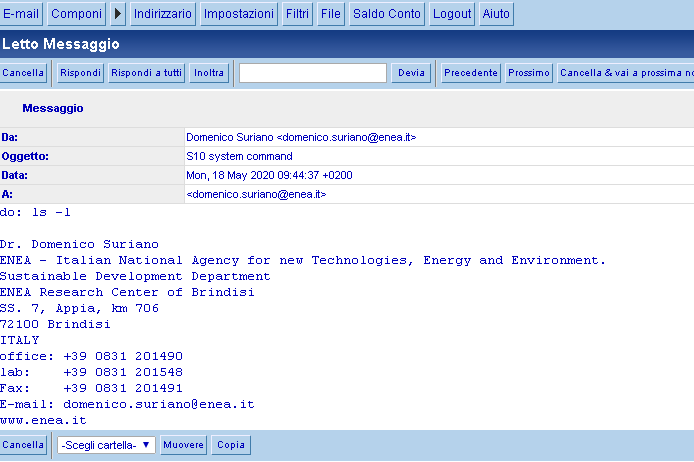


Figure 28: an e-mail containing the command for listing the files in the current working directory. In this case, the command will be executed by the operative system.


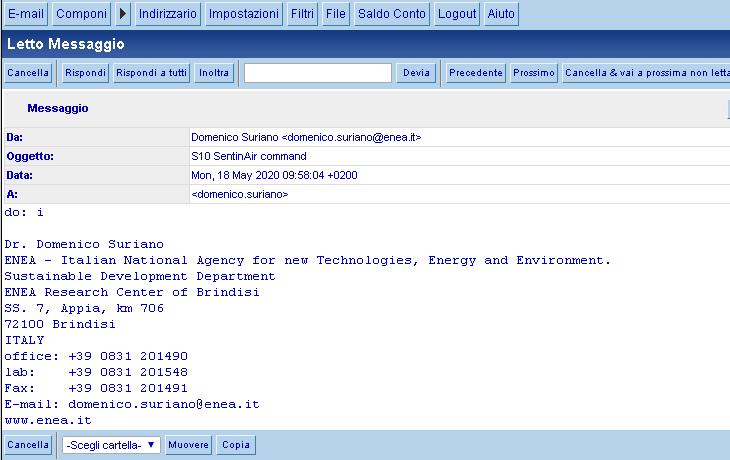


Figure 29: an e-mail containing a command for the SentinAir software.

*6.5 Expanding the SentinAir software with new device drivers*

The system presented in this work, as it is, can use all the devices listed in Table 1. Considering that each sensor or device featured by an analog output can be optionally connected to the LCSS adapter, or to the ADC PI board, which drivers are already included in the system, results that a wide range of devices can be currently used without developing new drivers. Anyway, by following the effort to provide the maximum flexibility and openness to the system, it has been planned that users having skills in Python programming will be enabled to develop new drivers to include in the pre-existing software. Of course, new drivers must be installed in the system by using the sentinair system installer (see section 6.2). The choice to adopt Python as the language for developing the SentinAir software is derived from the consideration that it has become one of the most popular programming languages [34], and it is also supported by a wide developer community that lowers the initial effort to learn it. Details and examples regarding the creation of new drivers can be found in the SentinAir user guide [20].

*6.6 Shutting down the system*

The device cannot be turned off by simply unplugging the power cable. The operative system installed on the mainboard, the software modules, and services running on it, need to be closed correctly. The system in its basic hardware configuration must be shut down by logging into it (as already explained earlier) and launching the command *'sudo halt'*. After executing this command, the user must wait few seconds (roughly 7 seconds), and therefore, the power cable can be unplugged from the socket. The typical hardware configuration offers an optional and quicker solution for shutting down the system: by holding pressed the button mounted on the enclosure lid, the yellow light starts to blink. After few seconds, all the three check lights turn off; this is the signal indicating that the system has finished the shutdown procedure and the button can be released. Therefore, at this point, the power cable can be unplugged.

1. **Validation and Characterization**

SentinAir system was tested both as a tool for calibration and evaluation of various sensors (see [10]), and as a portable monitoring unit (see [11]). To test the flexibility and the reliability of the system in different situations, various types of sensors and instruments were involved in these experiments.

*7.1 System test as a portable monitoring unit*

The system was involved in the POREM-LIFE17 ENV/IT/333 project [35] for gas emission monitoring purposes in a harsh and uncomfortable environment. This project is focused on proving the restoration capacity of repeated applications of poultry manure (properly treated) to selected soils in experimental zones. The site where these tests were performed is located in a rural and uncomfortable location far from the laboratory, featured by a weak and unstable internet wireless link.

The remote control of the device was performed through an “IP tunneling” service[28], but the wireless internet signal was very unstable and weak, for this reason, the data locally stored in the SD card were periodically downloaded also through the e-mail communication system, that provided the optional way to control the test advances.

During the development of the project, it was produced many hundreds of kilograms of treated poultry manure, which was stored in closed spaces; therefore, the system was tested for monitoring the concentration levels of NH_3_, H_2_S, CO_2_, and CH_4_. Two copies of the SentinAir device were used in this activity: the first one was placed in the depot where the manure heaps were stored, while the second one was placed just a few meters outside it. The monitoring activity was aimed to obtain indications about the impact of manure emissions by comparing the concentration levels detected by the two monitoring units. Both the devices were equipped with resistive sensors such as the TGS825 (H_2_S sensor by Figaro), the TGS826 (NH_3_ sensor by Figaro), and the TGS2611 (CH_4_ sensor by Figaro), which analog outputs were connected to the LCSS adapter. Ambient temperature and relative humidity were measured by the LCSS adapter onboard sensors, while the CO_2_ concentration was monitored by the IRC-A1 sensor by Alphasense. This last one is a Non-dispersive Infrared Radiation (NDIR) sensor usable with a support board provided by the manufacturer. This board is featured by a USB output that was connected to the SentinAir mainboard USB port. To complete the dataset featuring the poultry manure monitoring, a temperature probe inserted inside the heaps was also used for monitoring the internal temperature of the manure for tracking the progress of the fermentation process. The probe output, which is an analog signal, was connected to the LCSS adapter, while the device was deployed very close to the poultry manure heaps.

The sampling rate for both the devices was set to five minutes, and also, hourly and daily averages were computed in real-time by the system. Measurements lasted more than three months without any failure, proving that SentinAir is a useful and valid substitute for more expansive instrumentation that cannot be left in unattended and uncomfortable remote places. The experiment results are summarized in figure 30. The plots shown in this figure, highlight that the gas concentrations close to the manure and those outside the storage depot differ at least by one order of magnitude for each gas. This result gave us the indication that, if properly stored, the poultry manure has a limited impact on the surrounding environment.


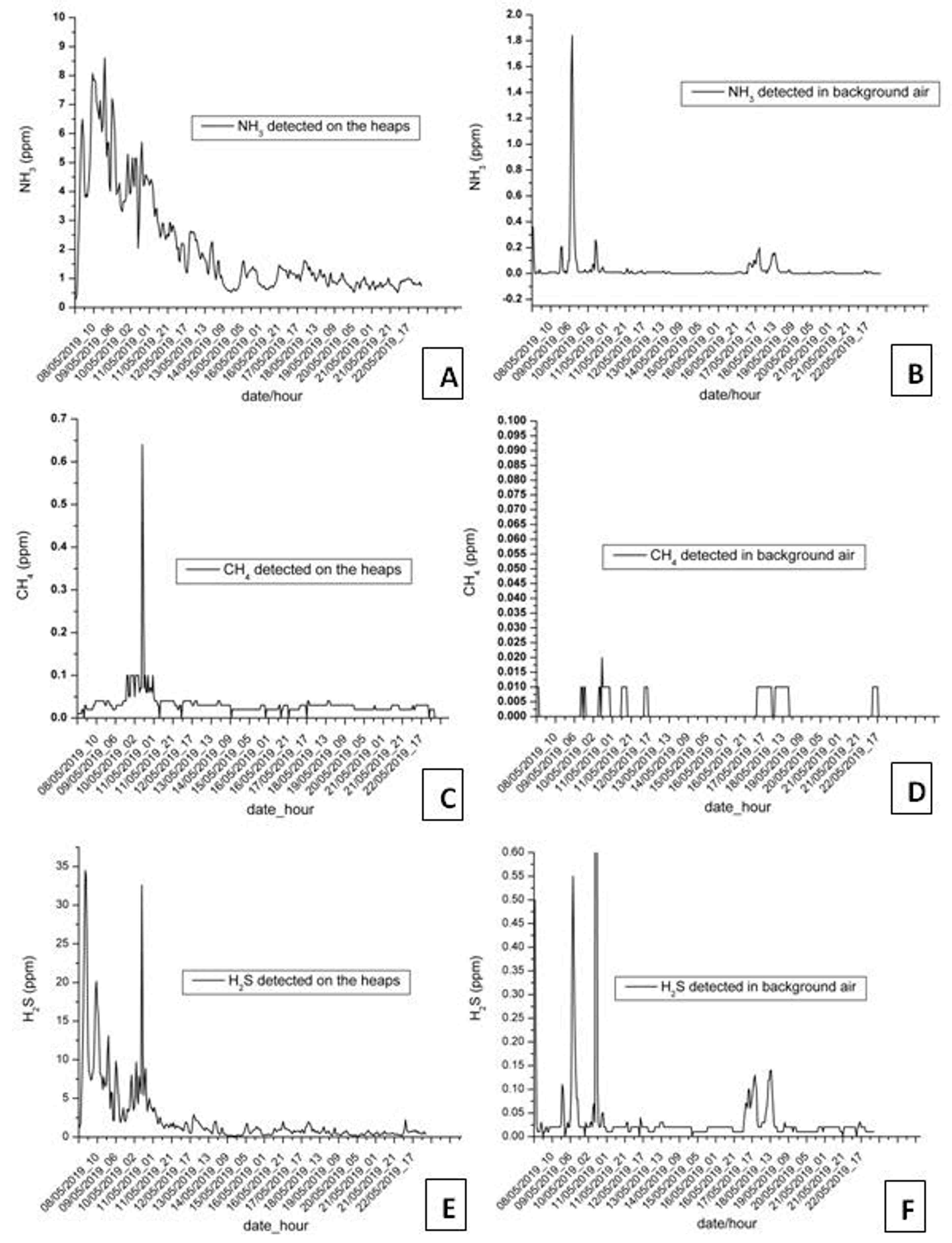


Figure 30: time series of the data acquired during the POREM project. Plots are referred to the five-minute sampling rate measures and report the first weeks of the experiment, which are the most significant: (A) NH_3_ measured close to the manure, (B) NH_3_ measured in background air,(C),(D),(E),(F) are related respectively to CH_4_ and H_2_S.

*7.2 System test as a tool for evaluation and calibration of sensors*

The functionalities of the system as a tool for facilitating the evaluation and the calibration of various low-cost sensors were tested by setting up two distinct experiments (see also [10]). In the first test, the system was used to evaluate the performances of two copies of the IRC-A1 sensor and also of two copies of the TDS0058 sensor by Dynament. These low-cost sensors are both designed for measuring CO_2_ concentrations, and their operating principle is based on the Non-Dispersive Infrared (NDIR) technology. As described previously, the IRC-A1 manufacturer provides also its electronic support board featured by a USB output, which gives the detected concentration of CO_2_ in ppm. Conversely, the TDS0058 sensor was mounted on the “Multisensor” board by Tecnosens, which provides both the sensor and the board ready-to-use. Even this board provides the CO_2_ concentration readings through the USB connection in ppm units. The dataset acquired in this experiment was also featured by temperature and relative humidity measures performed by the built-in sensors of the LCSS adapter. The evaluation of the sensors was performed by using a reference instrument, the 106L GO PRO by 2B technologies (see also table 1), that was connected to SentinAir through the USB port. Data coming out from the reference were included in the dataset stored in the on-purpose CSV file.

The site of the test was an office room where the SentinAir device and the reference instrument were placed in, while the data were downloaded through the WiFi LAN set up by the system.

The evaluation was performed starting from the CSV file where the dataset was recorded, and by computing the squared correlation coefficient (R^2^), the Mean Absolute Error (MAE), and the Standard Deviation (SD) as described in [10]. Figure 31 shows the time series of the data by comparing the sensor responses with the reference output, while table 5 summarizes the experiment results. By examining especially the R^2^ and MAE values, we can find similar performances in terms of comparison between the two types of sensors, and also in terms of difference between two copies of the same device. These elements also indicate that the sensor measures are in good agreement with the reference instrument readings, leading to the conclusion that the sensor outputs can be considered reliable.


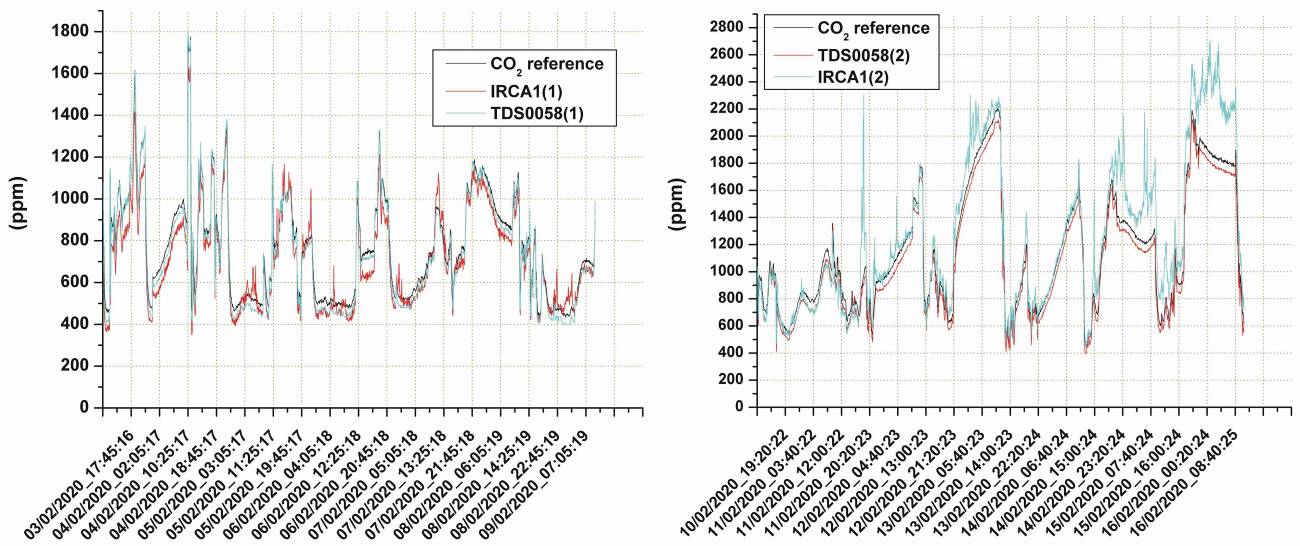


Figure 31: the time series that show the performance of the CO_2_ sensors during the tests for validating SentinAir as a tool for facilitating the low-cost sensor evaluation.

Table 5: results of CO_2_ sensors evaluation.

| **Sensor** | ***R^2^*** | ***MAE*** | ***SD*** | ***SLOPE*** | ***INTERCEPT*** |
| --- | --- | --- | --- | --- | --- |
| IRCA1(1) | 0,934 | 144,245 | 64,469 | 1,055 | 102,836 |
| TDS0058(1) | 0,985 | 121,211 | 32,277 | 0,954 | 153,947 |
| IRCA1(2) | 0,912 | 137,384 | 188,423 | 0,751 | 257,311 |
| TDS0058(2) | 0,995 | 119,128 | 32,25 | 0,985 | 134,328 |

A second test was set up to evaluate the system as a tool for facilitating the calibration of two different types of sensors designed for measuring ozone and nitrogen dioxide. As also described in [10], two copies for each of the NO2B43F (NO_2_ sensor), OXB431 (O_3_ sensor), and the SP-61 (O_3_ sensor) models were used in this experiment. The first two types of sensors involved in the test were electrochemical cells manufactured by Alphasense, while the last one was a semiconductor resistive sensor by Nissha-Fis. All of these models are featured by electronic boards supplied by the manufacturer that enable their ready use. These support boards provide an analog output, which is a voltage signal, related to the gas concentration sensed by the sensor. These outputs were connected to the LCSS adapter, which carried out the analog-to-digital conversions required to compose the dataset of the experiment. The LCSS adapter was also used to measure temperature and relative humidity, while the O342M and the AC32M by Environnement were selected as reference instruments. These last ones are featured by an internal UDP server providing a way to remotely control the instruments and the current measurements; therefore, they were connected to the SentinAir device through its Ethernet port.

The site selected for the test is located in an area belonging to the ENEA center of Brindisi, where a small room was used to host the two reference instruments, while the SentinAir device was placed outdoor, fixed to the external wall of the room. The experiment site was fairly out of the range of the WiFi signal emitted by SentinAir, therefore the control of the test was carried out through the “IP tunneling” service reachable by the internet connection.

The dataset contained in the CSV file recorded by the system was used to build the calibration equations for each sensor based on two different mathematical models: the linear regression, and the multivariate linear regression. Figures 32 and 33 show the time series that compare the measures of the reference instruments with the outputs provided by the sensors after the application of the two calibration models.


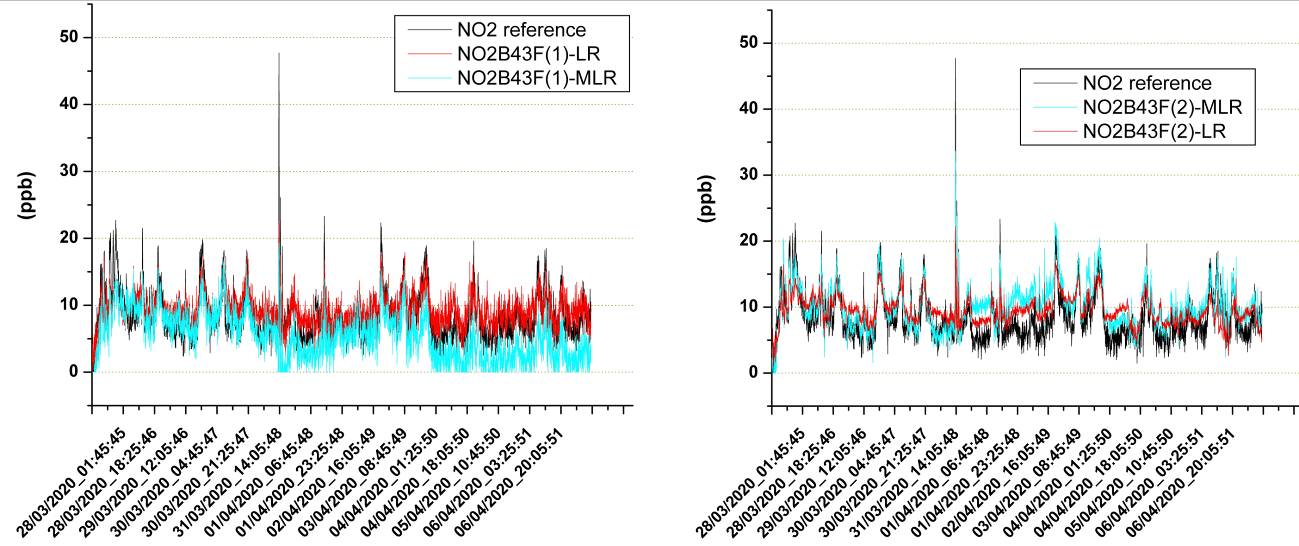


Figure 32: the time series given by the two copies of the NO2B43F sensor. The line labeled with NO2B43F-LR is related to the Linear Regression calibration model, while the other one is related to the Multivariate Linear Regression model.


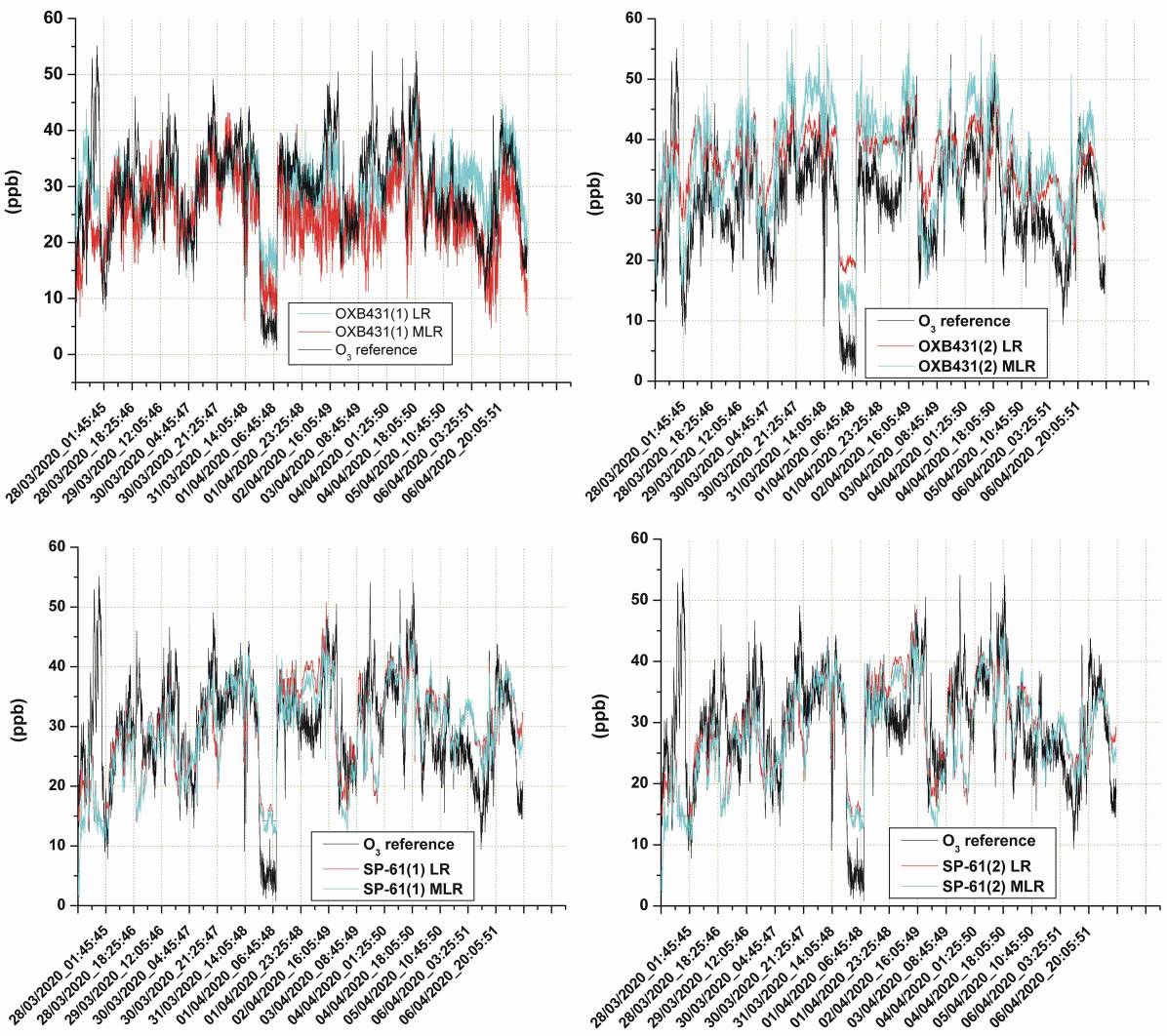


Figure 33: time series of ozone sensor outputs compared with the reference instruments. The LR labeled lines are related to the Linear Regression calibration model, while the MLR label indicates the output given by the Multivariate Linear Regression model.

1. **Conclusions**

Existing low-cost air quality monitoring units are mostly based on a fixed set of sensors and lack flexibility and openness. These factors bind to the use of a limited number of sensor types, typically those provided by the same manufacturer. Another limitation concerning many monitoring units is represented by the inability to be used in different environments or situations. The device presented in this document constitutes an effort to overcome these limitations. Thus, in this article, a low-cost device enabling the use of a wide range of sensors and regulatory-grade instruments at the same time in heterogeneous environments and situations has been proposed. The hardware of this system, along with its operating instructions has been illustrated, and its utility in different situations, such as rural and uncomfortable areas, indoor, or outdoor environments has been proved. It is highly expected that researchers having limited or notable experience in this area consider the use of this system in their research activities. By adopting open source solutions and providing several examples, it is also expected that more and more users will expand the software driver set of the sensors and instruments usable with this system. SentinAir is a project in continuous evolution. Improvements and updates can be found in the project repository [18].

1. **Acknowledgments**

This work was supported by funds of POREM (“Poultry manure based bioactivator for better soil management through bioremediation”) project co-funded by EU, within the LIFE program ([LIFE17/ENV/IT/333](https://www.sciencedirect.com/science/article/pii/S2352711020303022#GS1)).

1. **Declaration of interest**

Declarations of interest: none.

**References:**

[1] WHO, “Household air pollution and health”, *World Health Organization*, <https://www.who.int/en/news-room/fact-sheets/detail/household-air-pollution-and-health>, 2018, (accessed on 20 October 2020).

[2] Kampa M., Castanas E., “Human health effects of air pollution”, *Environmental Pollution,* 151(2)(2008), pp. 362-367, <https://doi.org/10.1016/j.envpol.2007.06.012>.

[3] McConnell R., Berhane K., Gilliland F., London S. J., Islam T., Gauderman W.J., et al., “Asthma in exercising children exposed to ozone: a cohort study”, *Lancet,* 359(9304)(2002), pp. 386–391, <https://doi.org/10.1016/S0140-6736(02)07597-9>.

[4] Castell N.,  Dauge F.R., Schneider P., Vogt M., Lerner U., Fishbain B., et al., “Can commercial low-cost sensor platforms contribute to air quality monitoring and exposure estimates?”, *Environment International*, 99(2017), pp. 293-302, <https://doi.org/10.1016/j.envint.2016.12.007>.

[5] Snyder E.G., Watkins T. H., Solomon P.A., Thoma E. D., Williams R. W., Hagler G. S. W., et al., “The Changing Paradigm of Air Pollution Monitoring”, *Environmental Science and Technology,* 47(20)(2013), pp. 11369-11377, <https://doi.org/10.1021/es4022602>.

[6] Karagulian F., Barbiere M., Kotsev A., Spinelle L., Gerboles M., Lagler F., et al., “Review of the Performance of Low-Cost Sensors for Air Quality Monitoring”, *Atmosphere*, 10(9)(2019), pp. 506-547, <https://doi.org/10.3390/atmos10090506>.

[7] Kumar P., Morawska L., Martani C., Biskos G., Neophytou M., Di Sabatino S., et al., “The rise of low-cost sensing for managing air pollution in cities”, *Environment International*, 75(2015), pp. 199–205, <https://doi.org/10.1016/j.envint.2014.11.019>.

[8] Lewis A., Edwards P., “Validate personal air-pollution sensors”, *Nature*, 535 (2016), pp. 29–31, <https://doi.org/10.1038/535029a>.

[9] Aleixandre M., Gerboles M., “Review of small commercial sensors for indicative monitoring of ambient gas”, *Chemical Engineering Transactions*, 30(2012), pp. 169–174, <https://doi.org/10.3303/CET1230029>

[10] Suriano D., Cassano G., Penza M., “Design and Development of a Flexible, Plug-and-Play, Cost-Effective Tool for on-Field Evaluation of Gas Sensors”, *Journal of Sensors,2020(2020),* Article ID 8812025, <https://doi.org/10.1155/2020/8812025>

[11] Suriano D., “SentinAir system software: a flexible tool for data acquisition from heterogeneous sensors and devices”, *SoftwareX,* 12(2020), Article ID 100589, <https://doi.org/10.1016/j.softx.2020.100589>

[12] Munir S., Mayfield M., Coca D., Jubb S. A., Osammor O., “Analysing the performance of low-cost air quality sensors, their drivers, relative benefits and calibration in cities—a case study in Sheffield”, *Environmental monitoring and assessment*, 191(94)(2019), <https://doi.org/10.1007/s10661-019-7231-8>

[13] Suriano D., Rossi R., Alvisi M., et al., “A portable sensor system for air pollution monitoring and malodours olfactometric control”, *Lecture notes in Electrical Engineering,* 109(2012), pp. 87-92, <https://doi.org/10.1007/978-1-4614-0935-9_15>

[14] Suriano D., Cassano G., Penza M., “A portable gas sensor system for air quality monitoring”, *Lecture notes in Electrical Engineering*, 268(2014), pp. 155-158, <https://doi.org/10.1007/978-3-319-00684-0_29>

[15] EarthSense UK website – Zephyr: <https://www.earthsense.co.uk/zephyr> (last access on 22 October 2020).

[16] Aeroqual air quality monitoring unit webpage: <https://www.aeroqual.com/outdoor-air-quality-monitors> (last access on 22 October 2020).

[17] Camprodon G., Gonzales O., Barberan V., Perez M., Smari V., de Heras A., Bizzotto A., "Smart Citizen Kit and Station: An open environmental monitoring system for citizen participation and scientific experimentation", *HardwareX,*6(2019), [https://doi.org/10.1016/j.ohx.2019.e00070](https://doi.org/10.1016/j.ohx.2019.e00070%20%20)

[18] SentinAir Github repository: <https://github.com/domenico-suriano/SentinAir> (last access on 22 October 2020).

[19] Scikit-learn libraries webpage: <https://scikit-learn.org/stable/> (last access on 22 October 2020).

[20] SentinAir user guide: [*https://github.com/domenico-suriano/SentinAir/blob/master/guide/sentinair-system-user-guide.pdf*](https://github.com/domenico-suriano/SentinAir/blob/master/guide/sentinair-system-user-guide.pdf) (last access on 22 October 2020).

[21] ADC Pi manufacturer website: <https://www.abelectronics.co.uk> (last access on 22 October 2020).

[22] Multisensor board supplier website: <https://www.tecnosens.it/en> (last access on 22 October 2020).

[23] Raspberry official website: <https://www.raspberrypi.org/> (last access on 22 October 2020).

[24] Raspberry operative systems webpage: <https://www.raspberrypi.org/software/operating-systems/> (last access on 22 October 2020).

[25] Device driver template webpage: <https://github.com/domenico-suriano/SentinAir/tree/master/devices/device-driver-templates> (last access on 30 November 2020).

[26] S. Raab, M.W. Chandra, “Mobile Ip technology and applications”, *Cisco Press*, 2005.

[27] A. Shneyderman, A. Casati, “Mobile VPN, delivering advanced services in next generation wireless systems”, *WileyPublishing Inc.*, 2003.

[28] Dataplicity website: [https://www.dataplicity.com](https://www.dataplicity.com/) (last access on 22 October 2020).

[29] Pitunnel website: <https://www.pitunnel.com> (last access on 22 October 2020).

[30] Remote.it website: <https://remote.it> (last access on 22 October 2020).

[31] Balena *Etcher download page*: <https://www.balena.io/etcher/> (last access on 2 February 2021).

[32] PuTTY website: <https://www.chiark.greenend.org.uk/~sgtatham/putty/> (last access on 22 October 2020).

[33] Online manual of “raspi-config” program: <https://www.raspberrypi.org/documentation/configuration/raspi-config.md> (last access on 2 February 2021).

[34] Perkel J.M., “Programming: Pick up python”, Nature, 518 (7537) (2015), p. 125 <http://www.nature.com/doifinder/10.1038/518125a>

[35] POREM-LIFE17 ENV/IT/333 website: [http://www.lifeporem.it/index.php/en/](http://www.lifeporem.it/index.php/en/%20) (last access on 22 October 2020).
